# Factors Affecting Hydrogen Adsorption in Metal–Organic Frameworks: A Short Review

**DOI:** 10.3390/nano11071638

**Published:** 2021-06-22

**Authors:** Vladimír Zeleňák, Ivan Saldan

**Affiliations:** 1 Department of Inorganic Chemistry, Faculty of Science, Pavol Jozef Šafárik University in Košice, Moyzesova 11, 04154 Košice, Slovakia; saldanivan@gmail.com; 2 Department of Physical and Colloid Chemistry, Faculty of Chemistry, Ivan Franko National University of Lviv, Kyryla and Mefodia 6, 79005 Lviv, Ukraine

**Keywords:** adsorption, hydrogen, MOF, nanoconfinement, metal hydrides

## Abstract

Metal–organic frameworks (MOFs) have significant potential for hydrogen storage. The main benefit of MOFs is their reversible and high-rate hydrogen adsorption process, whereas their biggest disadvantage is related to their operation at very low temperatures. In this study, we describe selected examples of MOF structures studied for hydrogen adsorption and different factors affecting hydrogen adsorption in MOFs. Approaches to improving hydrogen uptake are reviewed, including surface area and pore volume, in addition to the value of isosteric enthalpy of hydrogen adsorption. Nanoconfinement of metal hydrides inside MOFs is proposed as a new approach to hydrogen storage. Conclusions regarding MOFs with incorporated metal nanoparticles, which may be used as nanoscaffolds and/or H_2_ sorbents, are summarized as prospects for the near future.

## 1. Introduction

Creating an environmentally friendly and carbon-neutral society is currently a significant challenge. An emphasis on reducing the carbon footprint of human activities and efforts made to adopt sustainable and renewable energy sources resonate at various levels of society. Supported by funding of approximately EUR 1 billion, the European Commission has requested research and innovation projects aimed at responding to the climate crisis, and to help protect Europe’s unique ecosystems and biodiversity [1,2]. Numerous areas and activities exist in which society, politicians, researchers, and engineers are focusing on building a natural and more environmentally friendly economy. The transport sector and the automotive industry represent significant challenges. The introduction and use of batteries and electric vehicles is an important step toward reducing the carbon footprint of traffic; however, this strategy is applicable to urban transport rather than to freight, air, or shipping. Several large ocean-sailing container ships produce more harmful emissions than all the cars in the world combined. A solution to reduce greenhouse gases in various human activities, including heavy transport, is offered by hydrogen technologies, which have advanced considerably in recent years. Several economic forecasts indicate the approaching period of a hydrogen-based economy [3,4]. An example of this trend may be seen in the Olympic Games in Tokyo 2021 [5,6], where the Olympic torch is going to be powered by hydrogen, and Japan wants to use the Olympic games to promote hydrogen to the world [5,6]. Another sign of this trend is the anticipated growth of hydrogen technology companies’ share prices on the world’s stock exchanges [7].

The development of hydrogen technologies and a wider use of hydrogen fuel cell systems require new materials that can store large amounts of hydrogen at relatively low pressures with small volume, low weight, and fast kinetics for recharging. An ultimate system objective in 2020 for automobile fueling was set by the U.S. Department of Energy (DoE) at ~7 wt% hydrogen by weight [8]. Various materials have been studied in this respect, e.g., metal hydride systems [9,10,11,12,13,14,15,16,17,18]; however, numerous problems are associated with the use of high-temperature H_2_ storage materials (interstitial and complex metal hydrides or their reactive hydride composites), namely, their high cost, low specific uptake by weight, unfavorable kinetics requiring heating cycles, and susceptibility to contamination by impurities. In addition, various carbon-based adsorbents (carbon black, intercalated graphite, carbon nanotubes, and nanoporous polymer networks) have been widely studied and categorized as low temperature H_2_ storage materials, although they have been subject to ambiguous results [19,20,21,22,23,24,25,26,27]. It remains difficult to systematically engineer the molecular structure of these hydrogen adsorbers and identify specific hydrogen binding sites.

Among the most challenging materials for hydrogen storage are porous coordination polymers, also called metal–organic frameworks (MOFs). MOFs are two- or three-dimensional porous crystalline materials with infinite lattices. As a result of their ultra-high surface area values (more than 2500 m^2^·g^–1^ measured by the Brunauer–Emmett–Teller (BET) approach), they were found to be promising gas adsorbers for small gaseous molecules, including CH_4_, CHCl_3_, CCl_4_, C_6_H_6_, C_6_H_12_, CO_2_, Ar, N_2_, and H_2_. The main benefit of MOFs is their reversible and high-rate hydrogen adsorption process. A reasonable number of H_2_ molecules inside the body of MOFs may only be obtained at very low temperatures [28,29,30,31,32,33,34,35,36,37,38,39,40,41,42,43,44,45,46,47,48,49,50,51,52,53,54]. To date, MOFs have shown significant progress in applications of gas separation, catalysis, and coordination chemistry. For example, homochiral MOFs have become one of the most widely studied porous materials to enable enantioseparation [55]. Properties of zeolites and MOFs have been combined in metal–organic zeolites (MOZs) and proposed as promising catalysts [56]. The Cu-based boron imidazolate cage (BIF-29) with six exposed mononuclear Cu centers [57] and Ti-based MOFs (FIR-125, FIR-125, and FIR-127) made of Ti_8_O_8_(CO_2_)_16_ building units [58] have been considered to be effective photocatalytic materials. 

In the present review, we summarize the known physical and chemical factors affecting hydrogen adsorption in porous MOFs. We also propose new views. Thousands of different MOF structures are currently known, therefore, to clearly discuss the influence of the relevant factors of hydrogen adsorption, we focus mainly on MOFs that have cubic cavities of uniform size and internal structure. The focus is on the MOF-5 and its isorecticular MOF series (IRMOFs), that have the same framework topology and surface environment, but different pore sizes. When appropriate, we also discuss the known MOFs of other symmetries. Additionally, we provide a perspective of new MOF-based materials for effective hydrogen sorption. 

## 2. Basic Knowledge of Interaction between Solid Porous MOFs and Gaseous Hydrogen 

### 2.1. Hydrogen Sorption

Gas sorption and desorption (where the latter is the reverse of sorption) of the sorbate (e.g., gaseous hydrogen) by the sorbent (e.g., MOFs) is a dynamic process. Absorption occurs when the adsorbate may be incorporated into the internal structure of the adsorbent; hence, the structure and properties of the absorbate and absorbent may be modified. Absorption is often related to chemisorption, which occurs when the interaction force between the sorbent surface and the adsorbate is similar to that of chemical bonding in bulk compounds. Adsorption is a physical process of attracting atoms, ions, or molecules of the sorbate to the sorbent surface or interfacial layer and is related to weak intermolecular forces (van der Waals forces) of the same kind as those responsible for the nonideality of gases and the condensation of vapors. It is assumed that the electronic structures of sorbate and sorbent are not affected by physical adsorption. In the case of molecular H_2_ physisorption, the H–H bond in the gas phase is preserved in the sorbed state, whereas in the case of H_2_ chemisorption, the H–H bond is broken during the sorption process. Therefore, chemisorption can only occur in a monolayer on the sorbent surface, whereas physisorption is usually accompanied by multilayer adsorption. The heat values of hydrogen adsorption for most nanoporous materials, including MOFs, crosslinked polymers, or porous carbons, are within the range of 4–7 kJ·mol^–1^ [24].

### 2.2. Surface Area

According to IUPAC [59], porous materials are classified according to their pore sizes: macroporous (pore size > 500 Å), mesoporous (pore size ~20–500 Å), and microporous (pore size < 20 Å) materials. The microporous materials may be classified into supermicroporous (pore size ~7–20 Å) and ultramicroporous (pore size < 7Å). Most MOFs are microporous materials, although supermicroporous and ultramicroporous MOFs are well known. Ultramicropores are filled at very low relative pressures *p/p_o_* (where *p* is the equilibrium pressure and *p_o_* is the saturation vapor pressure at the adsorption temperature) directed by gas–solid interactions, and the rates of adsorption highly depend on temperature. In supermicropores, in addition to gas–solid interactions, a cooperation effect (when pore filling occurs at a relatively low *p/p_o_* value) takes place. Pore filling occurs when it is energetically as favorable for a gas molecule to exist between the monolayers of the gas in the center of the pore as it is to complete the monolayer coverage. A continuous monolayer of adsorbate molecules surrounding uniform sorbent surfaces is the main concept of the Langmuir adsorption model:

(1) $$\Theta_{A}=\frac{K_{eq}^{A}p_{A}}{1+K_{eq}^{A}p_{A}}$$

where *θ_A_* is the fractional occupancy of adsorption sites; *p_A_* is the partial pressure of the adsorbate; $K_{eq}^{A}$ is the equilibrium constant of the chemical interaction between the adsorbate molecule and the empty site. Most MOFs have different types of atomic surfaces; therefore, the interactions with gases highly depend on the metal atoms and organic ligands forming MOFs. At the evaluation of the MOF surface area, a common Langmuir equation may not be suitable. This is mainly due to localized adsorptions in ultramicropores and multilayer adsorption in supermicropores of MOFs.

The BET theory considers multilayer adsorption; therefore, this approach has become a common and simple method to calculate the surface area:

(2) $$\frac{p/p_{0}}{n\left(1-p/p_{0}\right)}=\frac{1}{n_{m}C}+\frac{\left(C-1\right)\left(p/p_{0}\right)\;}{n_{m}C}\;$$

where *n* is the specific amount of the adsorbed gas at the relative pressure of *p/p_o_*, *C* is the BET constant, and *n_m_* is the specific monolayer capacity. It has been shown that the BET surface area calculated from the N_2_ adsorption isotherm obtained by the Grand Canonical Monte Carlo (GCMC) simulation is very similar to the experimental BET surface area [60]. Reasonable values of the BET surface areas; however, may only be obtained in a linear range of Equation (2). In addition, this condition was experimentally confirmed mainly using *p/p_o_* values of 0.05–0.3, which are only applicable to non-porous or mesoporous materials. In the case of supermicroporous MOFs and ultramicroporous materials with pores that vary in terms of size, form, and chemical surroundings, the BET calculation on the surface area is inaccurate. In 2007, Llewellyn et al. recommended consistent criteria to address this issue [61]: (1) the BET approach should be limited to the range in which the term *n(1 − p/p_o_)* increases continuously with the *p/p_o_* value; (2) the *C* constant resulting from the linear fit should be positive with a value of at least 10; and (3) the *n_m_* value corresponds to a certain value of *p/p_o_*, which must be located within the linear region chosen for the area calculation. I. Senkovska and S. Kaskel [62] concluded that strong surface tension effects during subcritical adsorption can cause deformation of adsorbent structure, especially in the case of MOFs in which the tension forces act simultaneously when the pore is being filled. As a result, that the calculated surface area of the framework depends on the pressure range used in the calculation, the BET equation to determine the surface area of MOFs should be adapted to a specific situation. The specific surface areas of MOFs are generally determined from the N_2_ adsorption isotherms at 77 K; however, it should be mentioned that the use of alternative probe molecules will often result in different values of surface area.

In monolayer adsorption, all adsorbed H_2_ molecules are in contact with the sorbent surface. The monolayer can be in the form of different surface structures, including a close-packed array. The monolayer hydrogen capacity (*n_a,m_*) is determined as the number of H_2_ molecules sufficient to completely cover the sorbent surface. The surface coverage (*θ*) is assigned as the ratio of the adsorbed amount of H_2_ to the monolayer hydrogen capacity. The surface area (*A_s_*) of the sorbent is usually calculated from the monolayer hydrogen capacity when the area effectively occupied by the H_2_ molecules in the complete monolayer (*σ_m_*) is known:

(3) $$A_{s}=n_{a,m}\;L\;\sigma_{m}$$

where *L* is Avogadro’s constant. The specific surface area (*a_s_*) can be calculated as the ratio of *A_s_* to the mass (*m*) of the sorbent:

(4) $$a_{s}=\frac{A_{s}}{m}\;$$

### 2.3. Surface Excess and the Total Adsorbed Amount of Hydrogen

The adsorption process initially appears to be a simple procedure because, during the interaction between MOFs and the hydrogen gas, only the adsorption space exists (e.g., the adsorbed layer). In practice, however, to evaluate the total amount adsorbed (*n_a_*), the volume of the adsorption space (*V_a_*) is needed, which cannot be measured simply [60]. According to the Gibbs model, the difference in the amounts of the molecular hydrogen gas that would be present in the equivalent volume of the adsorbed phase in the presence and the absence of adsorption is a quantity called the surface excess amount *n_σ_*. In this case, adsorption is assumed to be two-dimensional (i.e., *V_a_* = 0) and to occur on the Gibbs dividing surface (GDS), which restricts the volume available for the homogeneous gas phase (*V_g_*). Calculating the gas phase amount (*n_g_*) in equilibrium with the adsorbent may be carried out with the appropriate gas laws. In practice, the *n_σ_* value may be determined by adsorption manometry or gravimetry. In the case of of H_2_ gas adsorption up to 1 bar, the *n_a_* and *n_σ_* values are nearly identical, provided the latter is calculated with the GDS very close to the adsorbent surface. When the H_2_ pressure >1 bar, the difference between the value of *n_a_* and *n_σ_* is clear. The experimental *n_σ_* data can be converted into the corresponding *n_a_* value, when the volume of the solid MOF (*V_s_*) and *V_a_* and are known. When the GDS exactly coincides with the actual adsorbent surface [63], the total adsorbed amount of hydrogen in the MOF is:

(5) $$n_{a}=n_{\sigma }+C_{g}V_{a}$$

where *n_a_* and *n_σ_* values are in mg·g^−1^; *C_g_* is the compressed gas concentration at a given temperature and pressure, in g·cm^–3^; and *V_a_* is the volume of the adsorption space in cm^3^. The *n_a_* value can be derived from the excess adsorption isotherm, which is the relationship between the *n_σ_* value and the equilibrium pressure of the gas (*p*) at constant temperature. At gas adsorption temperatures below the critical point, the *p/p_o_* value is usually considered, whereas at those above the critical point, only the *p* value must be considered, because no saturation vapor pressure (*p_o_*) exists.

The authors of [64] considered the volume of a pore as a complex object with a volume of the free-gas phase between two volumes of adsorbed molecules (*V_a_*). In a simplified case, the *V_a_* value is taken as a pore volume and may be calculated using the crystallographic density of the sample (*d_bulk_*) and the skeletal density of the material (*d_skeletal_*) [40]:

(6) $$V_{a}=\frac{1}{d_{bulk}}-\frac{1}{d_{skeletal}}$$

The obtained *V_a_* value is measured in cm^3^·g^–1^ and *d_skeletal_* is measured in g·cm^–3^:

(7) $$d_{skeletal}=\frac{m}{V_{skeletal}}$$

where *m* is the sample mass in grams and *V_skeletal_* is the skeletal volume of MOFs in cm^3^. *V_skeletal_* is normally determined using the gas sorption isotherm measured at 298 K up to the pressure of 100 bar. The hydrogen adsorption capacity in MOFs is expressed in wt% but more often the mass in mg or mmol of H_2_ adsorbed per gram of MOF is used [65].

### 2.4. Isosteric Enthalpy of Hydrogen Adsorption

Using the differential energies/enthalpies of adsorption (Δ*_ads_U*)/(Δ*_ads_H*) versus the adsorbed total amount (*n_a_*), the energetics of micropore filling can be estimated. The *Δa_ds_H* value at constant coverage can be evaluated by determining the isotherms at two or more different *T* values. The respective data processing may be related graphically as ln *P* for a given *n_a_* value as a function of 1/*T*. Using the Clausius-Clapeyron equation, it is possible to establish the “isometric heat of adsorption” when it is assumed that there is no enthalpy or entropy variation with *T* [66]:

(8) $$\Delta_{ads}H_{n}{}_{a}=R\;{\left(\frac{\partial \ln\left[P\right]}{\partial \frac{1}{T}}\right)}_{n_{a}}$$

where $\Delta_{ads}H_{n}{}_{a}$ is the enthalpy of differential adsorption and *R* is the gas constant. By comparison, using adsorption isotherms obtained at different temperatures, the energetics data assessment leads to the isosteric heat of hydrogen adsorption (*Q_st_*). As a result, that *Q_st_* is equal to −Δ*_ads_H*, the term “isosteric enthalpy of adsorption” is preferable [55]. Therefore, energy of hydrogen physisorption can be obtained directly from the hydrogen adsorption calorimetry (which is more reliable) or indirectly using the Clausius–Clapeyron relation:

(9) $$Q_{st}=R\ln\left(\frac{P_{1}}{P_{2}}\right)\frac{T_{1}T_{2}}{T_{2}-T_{1}}$$

The accuracy of this type of calculation depends on the measurement of the *P* values, which depends on the “adsorbent–adsorbate balance”. Typical problems appear in the case of gas adsorption in microporous materials at low *P* values. A small deviation in equilibrium due to molecular diffusion or thermal transfer can generate a relatively large variation in pressure measurements, leading to dispersion of the *Q_st_* value [66]. Evaluation of the *Q_st_* value has also been proposed using the Chakraborty–Saha–Koyama model [67] based on the concepts of chemical equilibrium potentials between the gaseous and adsorbed phases, the state equation, and the Maxwell relationships. The conventional form of *Q_st_* with the Clausius–Clapeyron approach was obtained as:

(10) $$Q_{st\;\left(conventional\right)}=RT^{2}\;\left[\;{\left(\frac{\partial \ln\left[P\right]}{\partial T}\right)}_{m_{a}}\right]$$

where *m_a_* is the adsorbate mass.

## 3. Structure and Topology of MOFs Studied for Hydrogen Adsorption 

### 3.1. MOF-5 and Isorecticular Compounds

The group of Yaghi described the first carboxylate-based MOFs, such as MOF-2 [68] and MOF-5 [69], in the mid-1990s. Subsequently, significant research interest has been shown in these materials, which have been tested for applications such as gas storage, separation, and conversion [70,71], and drug delivery [72]. As a result, several novel MOF crystal structures have been described, and thousands of different examples with various symmetries and topologies are known [68,69,70,71,72]. 

Due to the large number of existing MOFs and their structural diversity, in this work we focus mainly on MOFs with cubic symmetry, including the IRMOF series.

The well-known MOF-5 (also called IRMOF-1) was prepared under solvothermal conditions using the Zn^2+^ cation and 1.4-benzenedicarboxylic acid (H_2_BDC):

4 Zn^2+^ + 3 H_2_[O_2_C–C_6_H_4_–CO_2_] + 8 OH^–^ → 3 Zn_4_O[O_2_C–C_6_H_4_–CO_2_]_3_ + 7 H_2_O (11)

**Figure 1:**
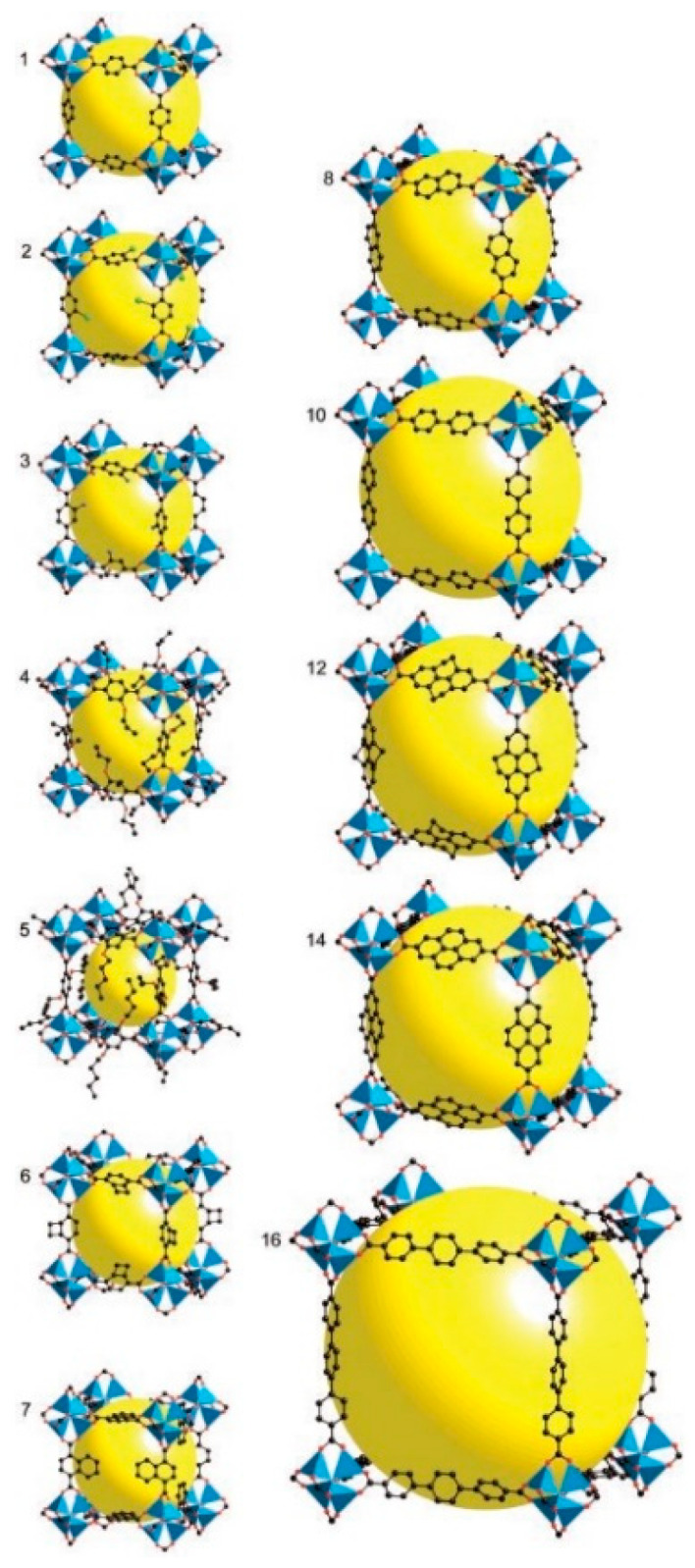
Isoreticular series of MOF-5 (IRMOF-1). Crystal structures of the IRMOFs-n (n = 1, 7, 8, 10, 12, 14, and 16), labeled respectively. The color scheme is as follows: Zn (blue polyhedra), O (red spheres), C (black spheres), Br (green spheres in 2), amino-groups (blue spheres in 3). The large yellow spheres represent the largest van der Waals spheres that would fit in the cavities without touching the frameworks. Reprinted with permission from [73]. Copyright © 2002, American Association for the Advancement of Science. All rights reserved.

This compound crystallizes in the *Fm-3m* space group with a cubic symmetry and composition of [Zn_4_O(BDC)_3_]_n_. The structure is formed by four Zn^2+^ cations that share one oxygen atom, forming regular tetrahedral [Zn_4_O]^6+^ clusters [73]. Each zinc atom is further tetrahedrally coordinated and each edge of the Zn tetrahedron is capped by a [BDC-CO_2_] group to form a [Zn_4_(O)(CO_2_)_6_] cluster [73]. These clusters are subsequently bridged by six BDC molecules in octahedral coordination to form a robust microporous cubic structure (Figure 1). Figure 1 shows one cavity of MOF-5—the overall structure is more complex, composed of larger cavities (15.1 Å in diameter) and smaller cavities (11.0 Å in diameter) in an alternating manner, so that the unit cell consists of four larger and four smaller cavities [69]. The calculated surface area of MOF-5 is about 2500–3000 m^2^·g^–1^.

The crystal structure of MOF-5 can also be described using topology tools. The structure can be viewed as a simple cubic six-connected net with two simplifications: (1) the nodes (vertices) of the net are replaced by clusters of the secondary building units (SBUs), which are Zn_4_O; and (2) the edges of the net are replaced by infinite rods (struts) of [–O_2_C–C_6_H_4_–CO_2_^–^] anions. MOF-5 is a prototype for a class of porous materials with similar structure constructed from octahedral [Zn–O–C] clusters made of organo-dicarboxylate linkers (struts), where “organo” represents, for example, biphenyl, tetrahydropyrene, pyrene, or terphenyl. Depending on the size of the acid molecule used, holes of different sizes can be prepared. These isostructural compounds are referred to as IRMOFs and have the same framework topology. Pore size in IRMOFs can be incrementally varied from 3.8 to 28.8 Å, and the open space can represent up to 91.1% of the crystal volume [73]. IRMOFs can be prepared with an organic linker substituted by –Br, –NH_2_, –OC_3_H_7_, –OC_5_H_11_, –C_2_H_4_, or –C_4_H_4_. From the IRMOF series, IRMOF-20, which is formed by thieno[3, 2-b]thiophene-2,5-dicarboxylate fragments, is of particular interest from a hydrogen adsorption perspective because, above 1 bar, IRMOF-20 shows higher gravimetric and volumetric hydrogen density than MOF-5, as shown by an isothermal pressure swing experiment between P_min_ = 5 bar and P_max_ = 35, 50, or 100 bar [74].

### 3.2. Other MOFs Formed by Metal Clusters Interconnected by Carboxylate Linkers

**Figure 2:**
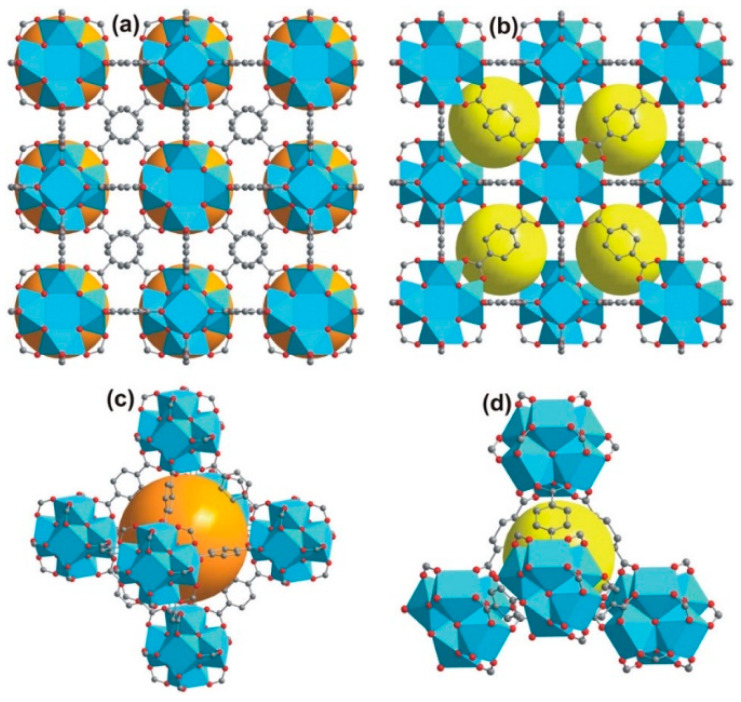
Ball-and-stick representations of the 3D cubic framework structure of UiO-66 (**a**,**b**). Parts of the framework showing spatial arrangements of the octahedral and the tetrahedral cages are represented by orange and yellow spheres, respectively (**c**,**d**). Magnified views of the octahedral and tetrahedral cages of Zr atoms are displayed as Zr (blue polyhedral), O (red spheres), and C (gray spheres). The hydrogen atoms and guest molecules have been removed from all the structural plots for clarity. Reprinted with permission from [76]. Copyright © 2013, Royal Society of Chemistry. All rights reserved.

Another type of MOF that may be promising for hydrogen adsorption is represented by a framework with a cubic structure based on metal clusters interconnected by polytopic carboxylate linkers. The well-known representatives of this MOF family are UiO (where UiO refers to the University of Oslo) and HKUST-1 (HKUST referring to the Hong Kong University of Science and Technology). UiO-66 is prepared from Zr^2+^ ions and [BDC]^2–^ ligands and crystalized in a cubic lattice with the *Fm-3m* space group, similar to MOF-5, and the SBU composition of [Zr_6_O_4_(OH)_4_(COO)_12_] (Figure 2a)[75,76].

**Figure 3:**
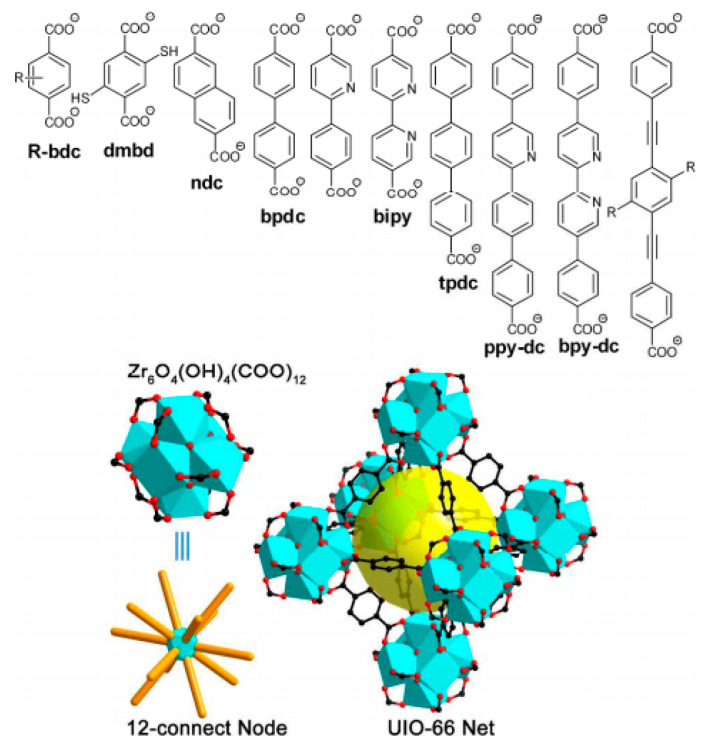
Representative ditopic carboxylate linkers used in the synthesis of the UiO frameworks. SBU with chemical composition of [Zr_6_O_4_(OH)_4_(COO)_12_] and the UiO-66 octahedral cage. The color scheme is as follows: Zr (blue polyhedra), O (red spheres), C (black spheres). The yellow sphere represent the largest van der Waals spheres that would fit in the cavity without touching the frameworks. Reprinted with permission from [77]. Copyright © 2014, Royal Society of Chemistry. All rights reserved.

The crystal structure of UiO-66 has two types of the cavities [76]: (1) an octahedral cavity with a size of ~9 Å (orange spheres in Figure 2a,c), and (2) a tetrahedral cavity with a size of ~7 Å (yellow spheres in Figure 2b,d). The UiO-66 material is unique because of its hydrothermal, mechanical, and thermal stability. The surface area value of UiO-66 is 1187 m^2^·g^–1^. By changing the [BDC] linker to a larger dicarboxylate ligand, an isorecticular UiO (Figure 3) can be prepared (e.g., UiO-67 [77], with surface area values within the range of 3000–4170 m^2^·g^–1^). In addition, other isorecticular MOFs derived from zirconium Zr_6_ clusters, present in UiO-66, have been synthesized (e.g., NU-1101, MOF-808, and PCN-521).

**Figure 4:**
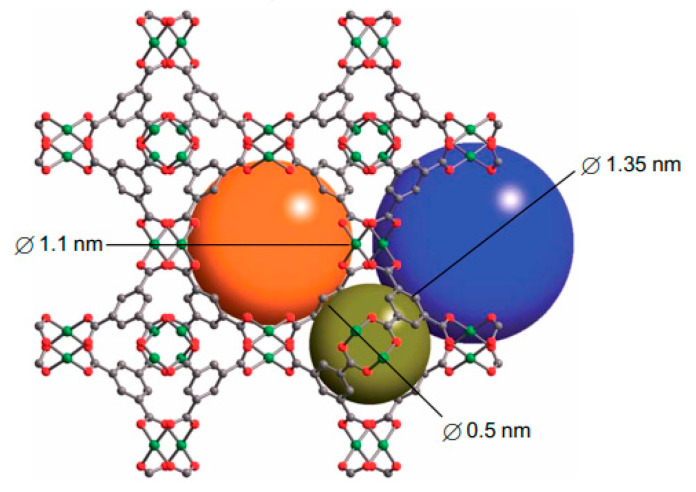
Crystal structure of HKUST-1 with three types of pores designated by differently colored spheres. Reprinted with permission from [79]. Copyright © The Royal Society of Chemistry 2014. All rights reserved.

The crystal structure of HKUST-1 with the composition of {[Cu_3_(BTC)_2_(H_2_O)_3_]}_n_ was first studied in 1999 [78]. It is composed of BTC^3-^ linkers (H_3_BTC = 1, 3, 5-benzenetricarboxylic acid), which coordinate with Cu^2+^ ions to form a cubic lattice with the *Fm-3m* space group. Within HKUST-1, the Cu^2+^ ions are organized in dimers with a “paddle wheel” cluster, in which each copper atom is coordinated by four oxygen atoms from the [BTC^3–^] bridging ligands. The fifth coordination site on each Cu^2+^ ion is occupied by an oxygen atom from a water molecule, which can be easily removed by activation. Coordination of [BTC^3–^] with the Cu^2+^ ions leads to the formation of a three-dimensional neutral skeleton with a surface area of 1500 m^2^·g^–1^ and a complex structure [79] with three types of pores (see Figure 4).

It should be noted that, in addition to the structure types described above, there are also other well-known MOF structures, such as the MIL and DUT families. A detailed structural description of these MOFs exceeds the scope of this review, and in the structure description we focus mainly on the structures with a cubic symmetry. In addition, we describe the structures of two other MOFs, namely, PCN-610/NU-100 and NU-1501, because these MOFs show significant hydrogen adsorption capacities. Ahmed et al. [48] undertook a systematic assessment of published databases of real and hypothetical MOFs, and screened nearly half a million metal–organic frameworks, which were examined computationally. The most promising materials identified computationally were subsequently synthesized and characterized experimentally. Importantly, three MOFs with usable capacities surpassing that of IRMOF-20 were demonstrated: SNU-70, UMCM-9, and PCN-610/NU-100, of which the latter showed the highest storage capacities.

**Figure 5:**
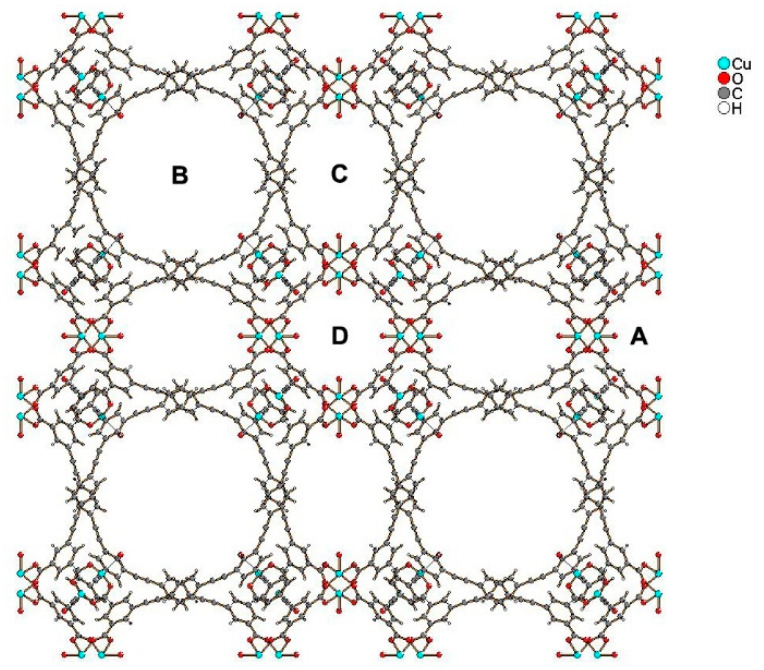
View of the structure of PCN-610/NU-100, where “A” shows paddle wheel Cu_2_(-COO)_4_ units, and “B”, “C”, and “D” show three types of voids of different sizes present in the structure.

Compound PCN-610/NU-100 contains ligands with C_3_ symmetry, namely 5,50,500-(((benzene-1,3,5-triyltris(ethyne-2,1-diyl))tris(benzene-4,1-diyl))tris-(ethyne-2,1-diyl))triisophthalic acid (H_6_ttei), with three coplanar isophthalate moieties. A solvothermal reaction of H_6_ttei and copper(II) nitrate in DMF/HBF_4_ yielded Cu_3_(H_2_O)_3_(ttei)⋅19H_2_O⋅22DMF, whose X-ray crystal structure revealed cubic *Fm-3m* symmetry with a noninterpenetrating (3,24)-connected framework formed from Cu_2_-paddle-wheel units (see symbol “A” in Figure 5). Interconnection of these paddle wheel Cu_2_(-COO)_4_ units with the H_6_ttei ligand results in formation of three types of voids (see symbols “B”, “C”, and “D” in Figure 5) with sizes 26.0, 18.6, and 12.0 Å, respectively. These voids can be described as cuboctahedra, truncated tetrahedra, and truncated octahedra [48].

**Figure 6:**
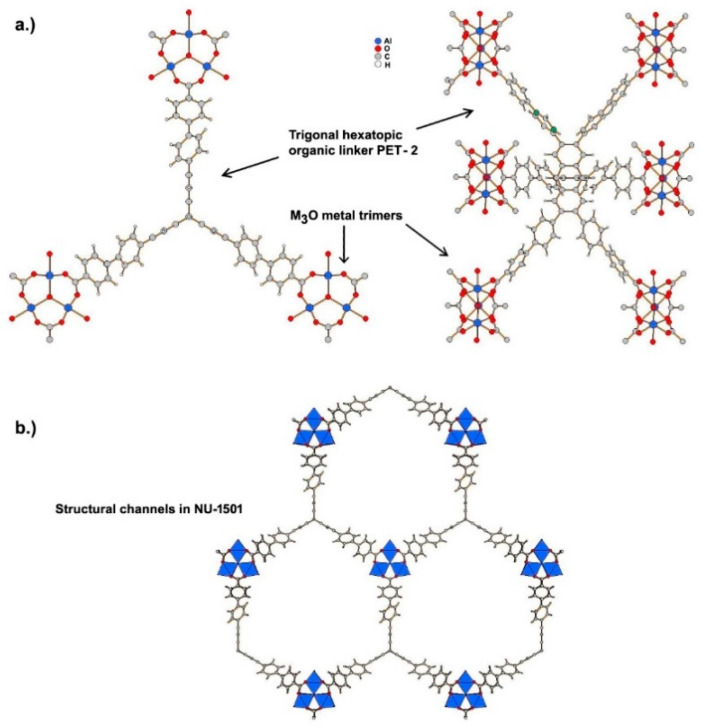
(**a**) Coordination of peripherally extended triptycene (H_6_PET-2) to metal cations forming M_3_O metal trimers. (**b**) Structural channels in NU-1501 viewed along *c* axis.

Ultraporous MOFs, namely NU-1501-M (M = Al or Fe), based on metal trinuclear clusters, have been reported [80]. The high porosity and surface area of these MOFs yielded impressive gravimetric and volumetric storage performances for hydrogen and methane. In contrast to the compounds described above, the structure of these MOFs has hexagonal rather than cubic symmetry, and the space group $P\bar{6}m2$. The compound is founded on the prismatic triptycene-based organic ligand, peripherally extended triptycene (H_6_PET-2), see Figure 6. The combination of these rigid trigonal prismatic linkers and M_3_O metal trimers (M = Al^3+^, Fe^3+^) forms MOFs with the formula [Al_3_(m_3–_O)(H_2_O)_2_(OH)(PET–2)] [80]. The compound exhibits 6-c acs topology, and have one type of open hexagonal channel with a pore size of ~2.2 nm (see Figure 6).

## 4. Current Approaches to Increasing Hydrogen Adsorption in MOFs

Unfortunately, no MOFs currently satisfy the proposed DoE target under ambient conditions. The porous MOFs adsorb molecular hydrogen physically; therefore, H_2_ adsorption capacity sharply decreases at higher temperatures. To predict hydrogen adsorption isotherms, GCMC simulations over a wide range of pressures at 77 K have been performed for a series of IRMOFs [81]. The obtained theoretical results suggest the existence of three adsorption modes at 77 K: (1) at low pressure (~1 bar of H_2_), the *n_a_* value is proportional to the value of *Q_st_*; (2) at ~30 bar of H_2_, the *n_a_* value is proportional to *A_s_*; and (3) at ~120 bar of H_2_, the *n_a_* value is proportional to *V_a_*. Due to the very weak adsorption energy between MOFs and H_2_ molecules, the correlations obtained for the total adsorbed amount of hydrogen at 77 K [81] could not be similarly exploited for those at 298 K [82]. Even at low *P*(H_2_) at 298 K, the *n_a_* value mainly correlates with the *V_a_* value, whereas the *n_σ_* value again correlates well with *Q_st_*. At high pressure, the *n_a_* value correlates better with *A_s_* than with *V_a_*. The authors of [81,82] predicted that a reasonable value of H_2_ adsorption capacity, such as 9 wt% of H_2_, could be obtained with MOFs that can provide the value of *Q_st_* within the range of 15–25 kJ·mol^–1^ with the *V_a_* value of ~2.5 cm^3^·g^–1^ and porosity of ~85%.

For MOF-5, the measured adsorption isotherm at 77 K showed type I behavior [59] with H_2_ adsorption capacity of 4.7 wt% of H_2_ at *P*(H_2_) = 50 bar [83]. The experimentally observed fast hydrogen adsorption at low hydrogen pressure indicates favorable sorption interactions between the MOF-5 and H_2_ molecules [28]. The hydrogen adsorption isotherm at 298 K was approximately linear because the MOF-5 framework was under-saturated with gas within the range of 5–20 bar of H_2_. Recently, it was found that the ultramicroporous MOF, NU-1501-Al, exhibits high values for both gravimetric and volumetric BET areas (7310 m^2^·g^–1^ and 2060 m^2^·cm^−3^, respectively [80]). These were considered the main reasons for the high value of hydrogen storage capacity (14.0 wt% and 46.2 g·dm^−3^) under a combined *T* and *P*(H_2_) change from 77 K and 100 bar to 160 K and 5 bar.

### 4.1. Factor of the Surface Area and Pore Volume

A linear relationship between the H_2_ adsorption capacity at 77 K and the *A_s_* value has been confirmed in numerous publications [40]. As a result of the *V_a_* value being proportional to *A_s_*, higher values of both *V_a_* and *A_s_* are recommended to improve hydrogen adsorption in MOF at 77 K. The adsorption capacity at 77 K and 1 bar of H_2_ is related to the *A_s_* values within the range of 100–2000 m^2^·g^–1^ and is not correlated in the case of higher *A_s_* values. This may be because the sorbent surface cannot be fully covered by H_2_ molecules at these high *A_s_* and low *P*(H_2_) values. In addition, it is most likely that the H_2_ molecules will preferentially bind on the most thermodynamically favorable sites in MOFs with the largest affinity to hydrogen. In the porous MOFs at a low pressure of 77 K, H_2_ adsorption may be affected by ligand functionalization, catenation, open metal sites, and pore size, whereas high-pressure H_2_ adsorption is directly proportional to the *A_s_* value.

The scaffolding-like nature of MOF-5 and IRMOFs results in remarkably high values of *A_s_* (2500–3000 m^2^·g^–1^ [28]). The crystal structure of MOF-5 serves as an ideal 3D location to adsorb hydrogen gas because the organic linkers are isolated from each other and accessible from all sides to adsorb the H_2_ molecules. MOF-5 samples prepared with different characteristics (low crystallinity, high crystallinity, interwoven, and interwoven with incorporated MWCNTs) have been studied to evaluate their effects on pore characteristics (determined by the BET approach for N_2_ adsorption at 77 K and *p/p_o_* value of 0.98) and H_2_ adsorption (measured at 77 K and 1 bar of H_2_) [84]. The obtained results confirmed that higher crystallinity in MOF-5 leads to higher *A_s_* and *V_a_* values, in addition to higher H_2_ adsorption capacity and thermal stability (Table 1). The interwoven MOF-5 samples showed an ultramicroporous structure and reached 1.7 and 2.0 H_2_ wt%, and an increase in the decomposition temperature, *T_dec_*, to 773 and 783 K, respectively.

The factors of surface area and pore volume are well recognized to have an impact on hydrogen adsorption. MOFs with very large surface areas usually show the highest hydrogen adsorption capacities. Typically, MOF materials with high BET areas, e.g., MOF-210 [82] and DUT-60 [85], show very high gravimetric adsorption capacities. Alternatively, as reported recently, balancing volumetric and gravimetric uptake must be considered for the development of ideal hydrogen adsorbents [80].

#### 4.1.1. Length of Organic Linkers

Previous research has shown that, for a given crystal structure of MOFs, the *A_s_* value can be increased using longer ligands (linkers). For example, BBC (4,4′,4″-[benzene-1,3,5-trilytris(benzene-4,1-diyl)]tribenzoate) may be considered to a longer model of BTB (4,4′,4″-benzene-1,3,5-triyl-tribenzoate). MOF-200, which comprises the BBC linker, demonstrates an *A_s_* value of 4520 m^2^·g^–1^, and MOF-177 with the BTB linker has an *A_s_* value of 4750 m^2^·g^–1^ (both using the BET method) [86]. A thorough comparison of isostructural UiO-66 and UiO-67 was made in [87]. These MOFs are constructed from Zr_6_O_4_(OH)_4_ nodes, and organic linkers 1,4-benzene-dicarboxylate and 4,4′-biphenyldicarboxylate for UiO-66 and UiO-67, respectively. Resulting from of the longer linker in the case of UiO-67, the value of *A_s_* (Langmuir) was 2483 m^2^·g^–1^, which is higher than that value of 1281 m^2^·g^–1^ in the case of UiO-66. A similar effect was observed for the *V_a_* value (0.85 vs. 0.43 cm^3^·g^–1^). As a consequence, the hydrogen adsorption capacity measured at 77 K and 38 bar of H_2_ was almost twice as high for UiO-67 (4.6 wt%) as for UiO-66 (2.4 wt%). A “ligand-truncation” strategy was studied in [88]. The idea was to reduce the *C_3_*-symmetry of the H_6_PHB linker (3,3′,3″,5,5′,5″-pyridyl-1,3,5-triylhexabenzoic acid) to the *C_2_*-symmetry of H_4_L (2,6-di(3′,5′-dicarboxylphenyl)pyridine) in GDMU-2. Therefore, this MOF changed the chemical composition from [Cu_4_(μ_2_–O)(PHB)_1_._5_(H_2_O)_2_]·(DMF)_5_(H_2_O)_4_ to {[Cu_2_(L)(H_2_O)_6_]_n_·4nDMF}. The new MOF with the H_4_L linker preserved the paddle wheel cluster, and the alignment of open metal sites and functional sites, similar to those observed in the GDMU-2. Hydrogen adsorption measured at 77 K and 1 bar of H_2_ for GDMU-2 was higher than that for the new MOF (240.7 vs. 193.0 cm^3^·g^–1^).

It was recommended that the increase in the *n_a_* value could be anticipated because IRMOFs have similar but larger organic linkers [28]. For comparison of such an organic linker function in IRMOFs, experimental data on pore characteristics and H_2_ adsorption collected in [40] are shown in Table 2.

The number of aromatic benzene rings may also influence the hydrogen sorption, as determined from the study of IRMOFs, in which the Zn_4_O(CO_2_)_6_ cluster is linked by chemically diverse organic linkers with a different number of benzene rings in the linker. The hydrogen sorption changed within the range of 0.89–1.73 wt% H_2_ [89]. In the case of MOF-177, which has a mixed (3,6)-connectivity due to the tritopic linkage of BTB, the hydrogen adsorption capacity was estimated to be 1.25 wt% H_2_. A crucial role of the organic linker was inconsistent with the model in which the metal oxide units dominate the sorption behavior in MOFs [90]. For example, IRMOF-20 and MOF-177 have a relatively low proportion of metal oxide to organic linker but, within the range of 70–80 bar H_2_, they demonstrated hydrogen adsorption of 6.7 and 7.5 wt%, respectively.

The results obtained for IRMOFs confirmed that the organic linker geometry, particularly the length, has a direct influence on the pore size, and hence the surface area of MOFs. In addition, when the *A_s_* values correlate with the *n_a_* values, the H_2_ adsorption capacity should depend on the length of the organic linkers. Therefore, the values of hydrogen adsorption capacity for IRMOF-6, IRMOF-8, IRMOF-11, and IRMOF-13 is considerably higher than that for IRMOF-1, IRMOF-2, IRMOF-9, and IRMOF-20 (Table 2). The elongation of the organic linker is limited, and may be collapsed in the case of the flexible MOF porous structure formed with long linkers. This behavior often leads to framework interpenetration, which results in a considerably reduced *A_s_* value.

#### 4.1.2. Catenation Process

From a thermodynamic perspective, the value of surface energy must be as low as possible to form a compact structure of the material. Therefore, when large pores in an MOF from a long organic linker are anticipated using only the geometrical calculation, an interweaving or interpenetration of the MOF framework may occur in practice. Interweaving and interpenetration are considered to be two types of the catenation process (bonding of atoms of the same element into a chain) where, respectively, a minimal and maximal displacement occurs between the catenated frameworks [91,92]. In an experimental work [93], it was proposed that catenation of MOFs may be controlled by adding a template during the solvothermal MOF synthesis (e.g., the oxalic acid used as a template promotes a non-catenated MOF framework). Using a brominated and non-brominated ligand, in which a non-catenated and catenated MOF framework was produced, the authors of [94] concluded that catenation may be controlled by the organic linker design. As a result of the synthesis of organic linkers and their organization in the MOF structure depending on the reaction parameters (e.g., the precursor concentration, temperature, and time of stirring), the catenation process in a different form is strongly guided by the MOF synthesis conditions [95,96].

It is obvious that catenation directly affects the H_2_ adsorption in MOFs. The copper paddle wheel node and the TATB linker (4,4′,4″-s-triazine2,4,6-triyltribenzoate) are the building units for both porous coordination networks (PCNs), catenated PCN-6 and non-catenated PCN-60, and their general formula is Cu_3_(TATB)_2_ [93]. After sample activation at 323 K, the hydrogen adsorption capacity at 77 K and 1 bar of H_2_ was 1.74 and 1.35 wt% for PCN-6 and PCN-60, whereas after activation at 423 K, these values increased to 1.9 and 1.62 wt%, respectively. The larger improvement in H_2_ adsorption for the non-catenated PCN-60 was explained by the open metal sites, which were blocked in the catenated PCN-6. Inelastic neutron scattering (INS) experiments suggested that in these MOFs, the open metal sites are most favorable for the H_2_ molecules, and at higher *P* values, the interaction between the H_2_ molecules and the organic linkers becomes stronger in the catenated PCN-6. The INS results were in good agreement with the high pressure H_2_ adsorption measurements at 77 or 298 K and 50 bar of H_2_ (Table 3). The experimental *n_a_* and *n_σ_* values confirmed higher H_2_ adsorption for the catenated PCN-6 than the non-catenated PCN-60. In addition to PCN-6 and PCN-60, high adsorption capacities were determined for the compound PCN-610/NU-100. Ahmed et al. [48] undertook a systematic assessment of published databases of real and hypothetical MOFs, and screened nearly half a million metal–organic frameworks, which were examined computationally. The most promising materials identified computationally were subsequently synthesized and characterized experimentally. For these three compounds, the authors reported hydrogen usable capacities for the pressure range of 5–100 bar. Of these compounds, the highest hydrogen usable gravimetric capacity was found for PCN-610/NU-100, with a value of 10.1 wt% [48].

Similar results were obtained for the catenated Zn_4_O(dcdEt)_3_ and Zn_4_O(dcbBn)_3_ (dcdEt=6,6′-dichloro-2,2′-diethoxy-1,1′-binaphthyl-4,4′-dibenzoate; dcbBn=6,6′-dichloro-2,2′-dibenzyloxy-1,1′-binaphthyl-4,4′-dibenzoate) [97]. Their four-fold interpenetrated frameworks adsorbed about 1.0 wt% of H_2_ at 298 K and *P*(H_2_) = 48 bar. This was explained by a smaller pore size, which increased the “guest–host” interactions. When MOFs are catenated too much, they show very low H_2_ adsorption. For example, the three-fold interpenetrated frameworks of [Cu_3_(4,4′-bpy)_1_._5_(2,6-NDC)_3_]_n_ ((bpy = bipyridine); NDC = naphthalenedicarboxylate)) and {[Cu(bpe)_0_._5_(2,6-NDC)]·0.5H_2_O}_n_ (bpe = 1,2-bis(4-pyridyl)ethane) showed low H_2_ adsorption capacities at 77 K and 15 bar of H_2_ (0.88 and 0.96 wt%, respectively) [98]. This may be due to negligible *A_s_* values estimated by the Langmuir method, i.e., 113 and 337.5 m^2^·g^–1^, for [Cu_3_(4,4′-bpy)_1_._5_(2,6-NDC)_3_]_n_ and {[Cu(bpe)_0_._5_(2,6-NDC)]·0.5H_2_O}_n_, respectively.

Therefore, using the catenation process, the pore characteristics may be altered, and hence the hydrogen adsorption capacity of MOFs can be changed. The authors of [99] concluded that the positive effect of catenation on H_2_ adsorption in MOFs is due to a strengthened framework and a smaller pore size.

#### 4.1.3. Different Organic Linkers

A combination of two types of organic linker within the same MOF structure could be another approach to increase the pore characteristics of MOFs. For example, the SNU-6 framework was constructed from the BPnDC (4,4′-benzophenone dicarboxylate) ligand and the bpy (4,4′-bipyridine) ligand, together with Cu^2+^ nodes [100]. This MOF was characterized by large pores (the pore diameter of 18.2 Å), and the *A_s_* values obtained by the BET and Langmuir methods were 2590 and 2910 m^2^·g^–1^, respectively. The hydrogen adsorption capacity at 77 K and 1 bar of H_2_ was relatively low (1.68 wt%); however, at high pressure (77 K and 70 bar of H_2_), the *n_a_* and *n_σ_* values were 10.0 and 4.87 wt%, respectively. The authors of the work [101] used coordination copolymerization of organic linkers with identical functional coordination but different topologies. BET and Langmuir *A_s_* values of 5200 and 6060 m^2^·g^–1^, respectively, were obtained for the UMCM-2 framework with the [Zn_4_O(T_2_DC)(BTB)_4/3_] formula (T_2_DC = thieno[3,2-b]thiophene-2,5-dicarboxylate). It should be noted that these high *A_s_* values originated from the novel framework topologies that were hard to obtain using only one type of organic linker. The experimentally obtained *n_σ_* value was around 6.9 wt% at 77 K and 46 bar of H_2_. In [102], the mixed BTB/NDC and BTE/BPnDC linkers (BTB = 4,4′,4″-benzene-1,3,5-triyl-tribenzoate; BTE = 4,4′,4″-[benzene-1,3,5-triyl-tris(ethyne-2,1-diyl)]tribenzoate) were used to obtain MOF-205 and MOF-210, respectively. These MOFs were characterized by high BET/Langmuir *A_s_* values (4460/6170 m^2^·g^–1^ and 6240/10,400 m^2^·g^–1^ for MOF-205 and MOF-210, respectively), which positively reflected on the H_2_ adsorption. For example, for MOF-210, the hydrogen adsorption capacity at 77 K was 8.6 wt% at 56 bar of H_2_ and 17.6 wt% at 80 bar of H_2_.

Approaches using different organic linkers have recently been developed with a mixed-matrix hybrid strategy to provide a tool for facile characterization of molecular transport in MOFs. For example, in [103], incorporation of the MOF crystals into polymers resulted in hybrid membranes with excellent molecular sieving properties. The improved membrane performance resulted from precise control of the organic linkers in the MOF, which delimited the entrance to the pores.

#### 4.1.4. Flexible Organic Linkers

**Figure 7:**
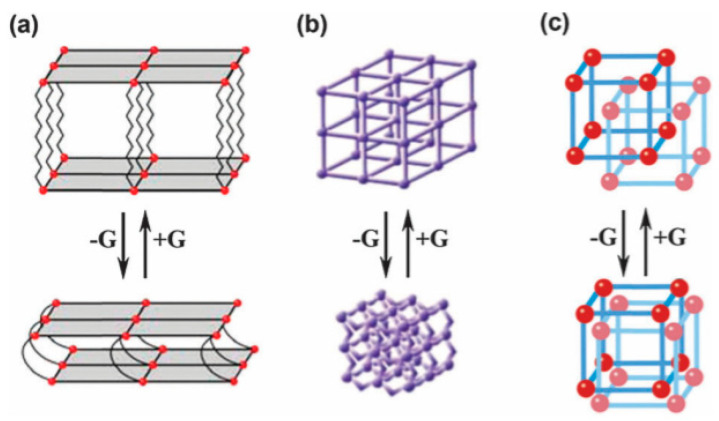
Three classes of breathing in dynamic 3D MOFs categorized by Kitawaga and Uemura. (**a**) In the case of pillared-layer MOFs, the reversible phases because of interlayer elongation and shortening could be realized using suitable flexible pillars. (**b**) For expanding and shrinking MOFs, they can show sponge-like dynamic behavior. (**c**) In interpenetrated grids which are densely packed in the absence of guests an introduction of molecules generates a sliding of one network. Reprinted with permission from [111]. Copyright © 2014, Royal Society of Chemistry. All rights reserved.

Since 2003, significant interest has been directed towards dynamic MOFs with flexible organic linkers [104,105,106,107,108,109,110]. Dynamic MOFs can respond to external conditions (e.g., *T*, *P*, electric or magnetic fields, and chemical insertion) and reversibly change their channels by a large magnitude while maintaining the same or similar topologies. Therefore, this type of MOF is often associated with reversible transformations between the expansion and the contraction states [105,106,107], which are called the “breathing” [108] or “sponge” [109] effect. For 3D dynamic MOFs, three situations have been distinguished (Figure 7) [111]. As a result of the interlayer extension and shortening, reversible transformations may occur using suitable flexible pillars (class a, Figure 7a). Class b corresponds to sponge-like dynamic behavior (Figure 7b). Finally, when interpenetrated high-packed frameworks occur (class c), the introduction of guest molecules promotes the sliding of one framework (Figure 7c).

**Figure 8:**
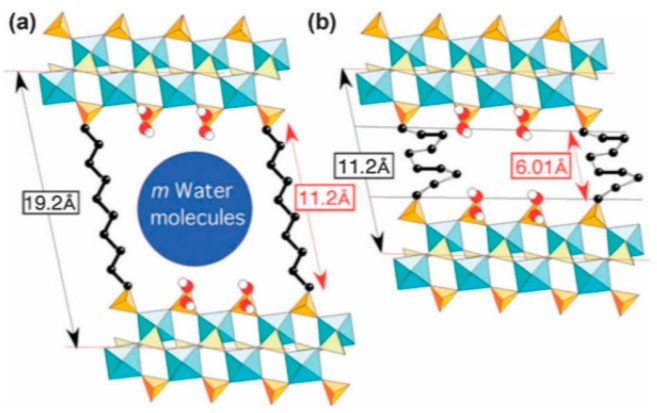
A schematic of the fully hydrated (**a**) and dehydrated (**b**) phosphate/diphosphonate showing the contraction of the carbon chain. Zr octahedra, colored in blue; phosphate tetrahedra, yellow; O, H, and C atoms are shown in red, white, and black, respectively. Reprinted with permission from [108]. Copyright © 2009, Royal Society of Chemistry. All rights reserved.

In the case of pillaring the layer using flexible linkers (class a), reversible extension and shortening of the pillars in the interlayers result in two stable MOF states. For example, in the layered γ–ZrPO_4_[O_2_P(OH)_2_]·2H_2_O frameworks, the [O_2_P(OH)_2_] groups can be substituted with different alkane diphosphonate ligands. The ZrPO_4_[O_2_P(OH)_2_]_1−*x*_ (O_2_POH–(CH_2_)*_n_*HOPO_2_)*_x_*_/2_·mH_2_O (*n* = 4–16; 0 < *x* < 1) materials, in which the degree of pillaring (*x*) was tuned by the reaction time, were synthesized in [106,109]. The reversible transformations were observed for low *x* values, where two different alkanediphosphonic chains were separated by the [O_2_P(OH)_2_] groups in the direction parallel to the layer. For example, fully hydrated 1,10-decanediphosphonate chains were extended (Figure 8a), whereas the dehydrated chains were shortened (Figure 8b). In practice, these transformations were reversible because the originally expanded framework could be prepared again via the chemical insertion of solvent molecules.

**Figure 9:**
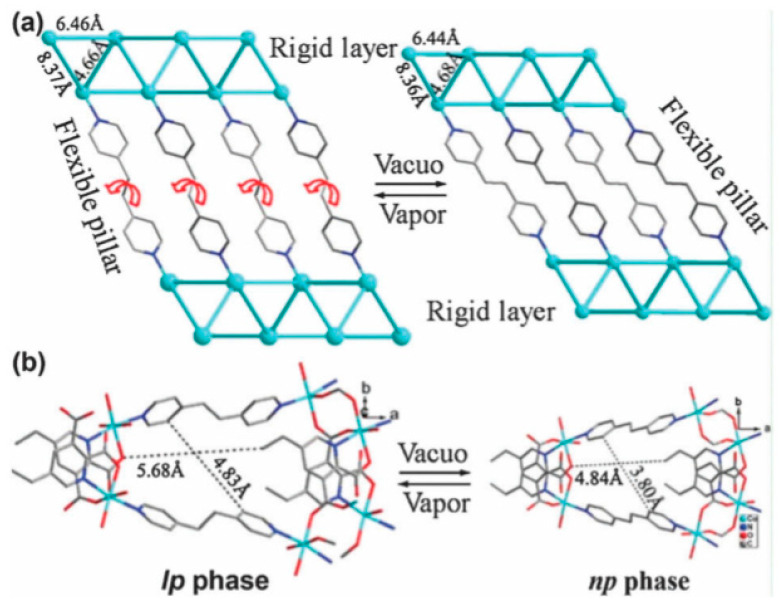
Structures of the pillared {[Co_2_(epda)_2_(etbipy)(H_2_O)_2_]·3H_2_O}_n_ framework (**a**) showing the rotation of the flexible ligand upon desolvation. The front view of the channels (**b**) showing reversible expansion (*lp* phase) and contraction (*np* phase) of the pores. Only Co atoms (blue balls) for each layer are shown for clarity. Reprinted with permission from [111]. Copyright © 2012, Royal Society of Chemistry. All rights reserved.

A pillared-layer {[Co_2_(epda)_2_(etbipy)(H_2_O)_2_]·3H_2_O}_n_ framework synthesized from H_2_epda (5-ethyl-pyridine-2,3-dicarboxylic acid) and etbipy (1,2-Bi(4-pyridyl)ethane) was introduced as a dynamic MOF in [111]. The framework formation was perfected by employing the rigid H_2_epda linker and Co^2+^ cations to form 2D networks, and the flexible etbipy linkers as pillaring ligands. The reversible extension and shortening of pillars were estimated to result in a 9% difference in the cell volume, while the crystal structure of expansion and contraction states remained unchanged (Figure 9). The decrease in the interlayer distance between the 2D Co-carboxylate layers from 15.77 to 14.35 Å was attributed to the rotation of the C–C single bonds of the flexible etbipy ligands, resulting in the relative gliding between the neighboring layers. The two pyridyl rings in the etbipy linker are almost perpendicular to each other in the expansion state, whereas they are almost coplanar in the contraction state.

A systemic synthesis and characterization of a series of MOFs with the pillared layer structure was carried out in [112]. The [Zn_4_(bpta)_2_(H_2_O)_2_] (H_4_bpta = 1,1′-biphenyl-2,2′,6,6′-tetracarboxylic acid) layers were connected by length-controllable bipyridine pillars. The authors developed a synthetic strategy that allowed systematic variation of the pillar to construct open frameworks with a similar structure. It was concluded that pore design could be adjusted by the selection of pillar ligands, hence leading to different hydrogen adsorption properties. The results obtained indicated that the activation process of these MOFs could affect the *Q_st_* value.

The hydrogen adsorption analysis of three flexible sulfur-containing MOF materials named M-URJC-n (M = Co, Cu, Zn) based on the 5,5′-thiodiisophthalic acid linker (H_4_TBTC) showed that these compounds display a gate-opening type adsorption mechanism at low pressures, attributed to the flexible nature of the ligand [113]. The hydrogen adsorption capacities of the compounds were not high, with levels of 2.81, 2.21, and 1.99 wt%, for Co–, Cu–, and Zn modification, respectively, at 77 K and up to 18 bar, and 0.12, 0.14, and 0.13 wt%, at 298 K and 170 bar, due to the presence of flexible ligands. These compounds showed an interesting gate-opening type adsorption mechanism. Considering the flexibility and the dynamic nature of the new structures, these compounds are candidates for applications such as hydrogen selective adsorption and gas separation processes, including hydrogen purification in precombustion mixtures of H_2_/CO_2_ [113].

A series of dynamic MOFs with ditopic organic linkers, which are the ligands capable of coordination at two separate sites and allow the creation of well-ordered extended complexes containing different cations, was prepared in [114,115,116,117,118,119]. The sponge-like dynamic mechanism is activated by rotation around the O–O axis of the carboxylate linker, which acts as a ‘‘kneecap’’ for the dynamic MOFs, either through a twisting or a bending mode. In combination with the integration of the ‘‘kneecap’’ into the interface between the metallic node and the organic linker, this approach may be used directly for the linkers themselves while keeping the MOF skeleton stable. In a recent review [120], synthesis of MOFs with flexible organic linkers was considered to be a promising means to shift H_2_ adsorption to higher pressures. Preparation of MOFs with the “breathing” effect, which gradually adsorb hydrogen gas with an increase in *P*(H_2_), can transfer physical sorption of H_2_ in MOFs significantly closer to the general requirements of hydrogen storage material.

### 4.2. Factor of Isosteric Enthalpy of Hydrogen Adsorption

Among MOFs with high *A_s_* values, some of the MOF examples have high values of hydrogen adsorption capacity, but only at cryogenic temperature. In practice, the hydrogen adsorption capacity at moderate temperatures designed by DoE falls to less than 1/10 of the value obtained at 77 K, and the *Q_st_* values in most porous MOFs is within the range of 5–10 kJ·mol^–1^ [121,122]. As a result of physical sorption of H_2_ molecules, van der Waals interaction between H_2_ molecules and the pore surface of MOFs is very weak. To increase the interaction at the ambient temperature, two recommendations have been made: (1) strong adsorption sites incorporated into the pores; and (2) optimization of the internal surface of MOF. In practice, creation of open metal sites, introduction of cations generating a strong electrostatic field within the cavities, doping with metal ions, infiltration of metal nanoparticles, and organic linker functionalization have been used to increase molecular hydrogen affinity to MOFs. In [123], it was concluded that MOFs can reach approximately 6 wt% H_2_ when they are characterized by a *Q_st_* value of 10–15 kJ·mol^–1^ and a free volume within the range of 1.6–2.4 cm^3^·g^–1^, or *Q_st_* > 20 kJ·mol^–1^ and a free volume smaller than 1.5 cm^3^·g^–1^. The authors of [124] studied H_2_ adsorption in an MOF at 298 K and 1.5 or 120 bar of H_2_, and measured deliverable capacity as the difference between the *n_a_* value at the high and the low hydrogen pressure. Their GCMC calculations suggested that for the maximum H_2_ delivery at 298 K, the optimal *Q_st_* value must be around ~20 kJ·mol^–1^. Other calculations [122] using the same strategy reached the conclusion that at 131 K and 1.5–100 bar of H_2_, the optimal *Q_st_* value should be ~6 kJ·mol^–1^. In [40], it was concluded that, even when the *Q_st_* values are increased by several units, they did not increase the high pressure H_2_ adsorption at 77 K, whereas at 298 K there is a tendency to form a proportional relationship between the *Q_st_* and the *n_σ_* values.

#### 4.2.1. Open Metal Sites at Secondary Building Units and Organic Linkers

It was shown that metal centers unsaturated in coordination, often called “open” or “accessible” metal sites, may increase the *Q_st_* value [125,126,127]. In practice, the H_2_ adsorption values measured at 298 K for MOFs with open metal sites are higher than those for MOFs without open metal sites. In addition, this tendency is pronounced for MOFs with a high *A_s_* value (>3000 m^2^·g^–1^). Based on experiments involving the introduction of open metal sites in MOFs, several possible approaches have been highlighted: (1) metal nodes with open metal sites; (2) metal clusters coordinating solvent molecules, in which the solvent is removable; (3) organometallic complexes connected with the aromatic organic linkers [126]; (4) metal complexes intercalated through electrostatic forces [126]; and (5) metal cations in the anionic frameworks [127].

Open metal sites in MOFs may be constructed of metal cluster SBUs coordinating solvent molecules, followed by removal of the solvent using thermal treatment. In this case, the SBUs are very often the bimetallic paddle wheel units of M_2_(O_2_CR)_4_ (M=Cu^2+^, Zn^2+^, Cd^2+^), in which solvent molecules are coordinated at the axial sites. The fluorite-like structure of the [Co^II^_4_(μ–OH_2_)_4_(MTB)_2_(H_2_O)_4_]_n_·13nDMF·11nH_2_O framework (SNU-15) promotes 3D channels in which every Co^2+^ ion coordinates the aqua ligand, and the Co-Co distances are 3.550 and 3.428 Å. When the coordinated H_2_O molecules are removed, the vacant coordination sites are created on the Co^2+^ ions. Due to the values of the Co–Co distance, H_2_ molecules can be bonded in a side-on manner that results in the *Q_st_* value of 15 kJ·mol^−1^ at zero coverage [124]. The same high *Q_st_* value was found for the [Co^II^_4_(μ-OH_2_)_4_(MTB)_2_]*_n_* (MTB = methanetetrabenzoate) framework (SNU-150′) prepared by heating the SNU-15 at 493 K under a vacuum for 24 h [128]. The synthesis of MOFs with entatic metal centers, in which it is possible to bind the substrate without ligand removal, may be considered another method to create the open metal sites via the coordination of the solvent molecules by the metal clusters. For example, PCN-9 with the H_2_[Co_4_O(TATB)_8/3_] formula (TATB = 4,4′,4″-s-triazine-2,4,6-triyltribenzoate) holds the square-planar SBU that is Co_4_(μ_4_-O) [129]. The Co atoms in the SBU are five-fold-coordinated with a square-pyramidal geometry, and the position below the square plane formed of the four O atoms is in an entatic state and ready to bind a molecule to achieve octahedral coordination. This square planar μ4-oxo bridge found in PCN-9 is a unique phenomenon in MOFs. The *A_s_* value, obtained by the Langmuir method, of 1355 m^2^·g^–1^, and the *V_a_* value of 0.51 cm^3^·g^–1^ were found for the desolvated PCN-9. Hydrogen adsorption at 77 K and 1 bar of H_2_ and the *Q_st_* values for the PCN-9 were 1.53 wt% and 10.1 kJ·mol^–1^, respectively. In a recent work [130], the MOFs M_2_(*m*-dobdc) and the isomeric frameworks M_2_(dobdc) (M = Co or Ni; *m*-dobdc4−=4,6-dioxido-1,3-benzenedicarboxylate) were evaluated for their volumetric hydrogen capacity. At 298 K, hydrogen storage of 11.0 g H_2_·L^–1^ and 23.0 g/L with a temperature change between 198 and 298 K were experimentally confirmed for Ni_2_(*m*-dobdc) within the range of 5–100 bar of H_2_, whereas the DoE target in 2020 was 30 g H_2_·L^–1^. These results were explained by the presence of open metal cation sites (highly polarizing Ni^2+^), which strongly interacted with H_2_ molecules, thus providing their dense packing within Ni_2_(*m*-dobdc).

The open metal sites at organic linkers are also a highly popular approach used to increase the *Q_st_* value. For example, the structures of MOFs constructed from various types of porphyrin complexes are very well known [131,132,133,134]. They contain open metal sites with respect to the square-planar coordination plane, therefore the porphyrin-based MOFs may be recommended for effective H_2_ adsorption [135,136,137,138]. The MOFs constructed from the Schiff base complexes are also characterized by open metal sites. A series of isorecticular chiral MOFs (IRCMOFs) from the chiral Mn-salen-derived dicarboxylate linker and [OZn_4_]^6+^ nodes containing the Mn^3+^ open metal sites were synthesized in [139]. As a result of the collapse of the framework during the activation process, the *A_s_* value of the IRCMOFs was very small, which most likely explains why data on H_2_ adsorption are lacking [140,141,142,143,144,145,146]. The approach in which metal fragments are attached to the organic linkers involves covalent functionalization, followed by metalation and attachment of organometallic complexes to the aromatic components of the linkers. The authors of [147] synthesized the metalated ligand [4,7-bis(4carboxylphenyl)-1,3-dimethylbenzimidazol-2-ylidene](pyridyl)Pd(II) iodide and then integrated it into an IRMOF, which was denoted IRMOF-77 within the [Zn_4_O(C_28_H_21_I_2_N_3_O_4_Pd)_3_] framework. From the H_2_bpydc linkers (2,2′-bipyridine-5,5′-dicarboxylic acid), they synthesized the [Al(OH)(bpydc)] framework, namely MOF-253, with open 2,2′-bpy coordination sites [148]. As a result of the accessibility of the chelating bpy units, PdCl_2_ and Cu(BF_4_)_2_ were impregnated inside the MOF from the solutions of PdCl_2_ and Cu(BF_4_)_2_ in acetonitrile. Therefore, MOF-253·xPdCl_2_ (x = 0.08; 0.83) and MOF-253·0.97 Cu(BF_4_)_2_ were prepared. In conjunction with the covalent and coordinate covalent postsynthetic modification, the MOF was covalently connected to a chelating group, and then the metalated group. The Zn-pillared paddle wheel MOF with Zn_2_(TCPB)(DPG) (TCPB = 1, 2, 4, 5-tetrakis(4-carboxyphenyl)-benzene; DPG = meso1,2-bis(4-pyridyl)-1,2-ethanediol) framework was synthesized and then reacted with the succinic anhydride in [149]. During the chemical reaction between diols in the MOF and succinic anhydride, a product with opened ring and free carboxylic acid groups was obtained. Post-synthetic modification of these carboxylic groups was carried out through the solution impregnation of CuCl_2_ from its aqueous solution. In [150], the metal-complexed MOF (Zn_4_O)_3_(BDC–C_6_H_5_N_2_PdCl_2_)_3_(BTB)_4_ was obtained by chemical reaction between the covalently bound iminopyridine chelate derivative (Zn_4_O)_3_(BDC–C_6_H_5_N_2_)_3_(BTB)_4_ and PdCl(CH_3_CN)_2_. In [151], Zn_4_O[(bdc)Cr(CO)_3_]_3_ was prepared through chemical interaction between the benzenoid phenyl rings of MOF-5 and the Cr(CO)_3_ groups. Then, the decarbonylated framework with three open coordination sites per metal was prepared by heat treatment at 473 K. As a result of the aggregation of Cr atoms, the low-pressure H_2_ adsorption at 298 K was lower than the 0.2 H_2_ molecules per formula unit of the MOF. Using the photodecomposition reaction, substitution of a single CO ligand per metal by the H_2_ molecule to give Zn_4_O[(bdc)Cr(CO)_2_(H_2_)]_3_ may be possible. Taking into account the hydrogen binding energy for [(C_6_H_6_)Cr(CO)_2_(H_2_)] and [(C_6_H_5_Me)Cr(CO)_2_(H_2_)], a *Q_st_* value of 60–70 kJ·mol^–1^ was calculated for Zn_4_O[(bdc)Cr(CO)_2_(H_2_)]_3_ [152]. MOFs with open metal sites at secondary building units and organic linkers are capable of binding several H_2_ molecules at a given binding site. This MOF strategy was considered one of the most effective means to increase H_2_ volumetric capacity in adsorbers of hydrogen gas [153].

#### 4.2.2. Metal Ions for an Electrostatic Field within the Cavities

Another approach to increasing the *Q_st_* value is to exchange the cations integrated in the anionic framework with metal ions that have higher affinity to H_2_ molecules. For example, in the Mn_3_[(Mn_4_Cl)_3_(BTT)_8_]_2_, (BTT = 1, 3, 5-benzenetristetrazolate) framework, Mn^2+^ ions can be exchanged with Li^+^, Cu^+^, Fe^2+^, Co^2+^, Ni^2+^, Cu^2+^, or Zn^2+^ ions [126]. Chemical compositions based on the relative ratio of Mn^+^/Mn^2+^ showed that the total number of extra framework cations is not higher than five. The *Q_st_* values, calculated using a virial fit to the H_2_ adsorption isotherms at 77 and 87 K, demonstrate a relatively large variation, but they deviate in almost the same region as those for the Mn_3_[(Mn_4_Cl)_3_(BTT)_8_]_2_ framework. The values of hydrogen adsorption capacity measured at 77 K and 1.2 bar of H_2_ for the ion-exchange frameworks and the original Mn_3_[(Mn_4_Cl)_3_(BTT)_8_]_2_ were within 2.00–2.29 wt%.

The authors of [154] declared that MOF-5 was partially doped with the Co^2+^ ions during crystallization in the solvothermal synthesis. Based on inductively coupled plasma (ICP) analysis, the prepared materials were considered to be Co8-MOF-5 with the composition of the Zn_3_._68_Co_0_._32_O(BDC)_3_(DEF)_0_._75_ (DEF = diethylformamide) framework containing 8% Co and 92% Zn, and Co21-MOF-5 with the composition of Zn_3_._16_Co_0_._84_O(BDC)_3_(DEF)_0_._47_ containing 21% Co and 79% Zn. The ICP results showed that for these Co-MOF-5 materials, the substitution of more than one Co^2+^ ion in [OZn_4_]^6+^ nodes was difficult. In addition, the XRD results confirmed that the crystal structure of both undoped and Co-doped MOF-5 was identical. In practice, during the removal of solvent, the coordination geometry around Co^2+^ changed from octahedral to tetrahedral due to the loss of two DEF molecules. The values of the hydrogen adsorption capacity at 77 K and 10 bar of H_2_ were in the order of Co21-MOF-5 > Co8-MOF-5 > MOF-5. A small increase in the *Q_st_* value was experimentally found for Co21-MOF-5 (∼7 kJ·mol^–1^ vs. 5.5 kJ·mol^–1^ for MOF-5). The results obtained suggested that the Co^2+^ ions incorporated into MOF-5 played an important role in the hydrogen adsorption process, even being incorporated into the “protected” metal sites that were less accessible to H_2_ molecules.

Recently, a theoretical assumption of a “regional dynamic electric field effect” was proposed in [155] to explain a selective adsorption of CO_2_ over N_2_, O_2_, and CH_4_ in the [Co(tipb)(adc)](DMF)_3_(H_2_O)_1_._5_ (tipb = 1,3,5-tris(p-imidazolylphenyl)benzene; adc = 9,10-anthracenedicarboxylate) framework. It was concluded that regional dynamic electric fields do exist in MOFs, and their polarizability and quadruple moment might be considered the crucial factors for gas adsorption. According to the proposed effect, the gas adsorption capacity and selectivity can be enhanced by intensifying the electric field and potential gradient of the effective adsorption space in the MOF through structure modification. This cobalt-based MOF with tipb and adc linkers is a good example of making a connection between peculiarities in the crystal structure, the level of H_2_ adsorption, and the *Q_st_* value. In practice, the metal doped MOFs demonstrated a positive impact on hydrogen adsorption at 298 K, which might be explained by a higher interaction between metal ions and H_2_ molecules.

### 4.3. Catalytic Effect and Pore Design

Due to their high affinity to hydrogen, the platinum group metals were the first catalysts used in various hydrogenation reactions and processes related to H_2_ molecules. Subsequently, a hydrogen spill-over effect was defined as dissociative chemical sorption (i.e., H_2_→2H) on the catalyst surface and the later migration of H atoms onto the surface of support. The catalytic effect was also used for MOFs in an attempt to accelerate the hydrogen adsorption process. In addition, the morphology of pores and their extra functionalization were taken into consideration. In the case of microporous MOFs, the morphology of the pores represents their main characteristics, including the geometrical shape (pore width and pore volume) and roughness of the pore walls. A small pore diameter was found to be an important factor for the achievement of a high value of hydrogen adsorption capacity at room temperature.

#### 4.3.1. Incorporated Metal Nanoparticles

**Figure 10:**
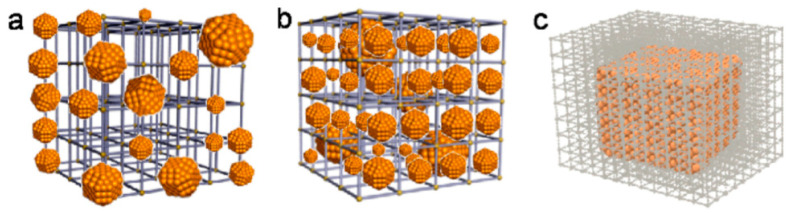
Representative methods of preparation of composites consisting of metal nanoparticles and MOF. (**a**) A solid grinding method. (**b**) The solution infiltration. (**c**) An encapsulation of presynthesized nanoparticles by growing the MOF around them. Reprinted with permission from [158]. Copyright © 2016, American Chemical Society. All rights reserved.

Recently, the idea of the hydrogen spillover effect has been transferred to MOFs, and the introduction of several types of metal nanoparticles (NPs) to MOFs has been considered (Figure 10) [156,157,158,159]. The simplest method involves preparing a coating made of the NP metal on the MOF surface, but without completely wrapping the MOF in a metal thin film (Figure 10a). Using a solid grinding approach, Au NPs were attached to the surface of the well-known MOF examples, including MOF-5 [160]. The solution impregnation method was reported to be a convenient tool for preparing metal NPs infiltrated inside MOFs (Figure 10b). Typically, the metal precursor solution fills the MOF pores by capillary force, and the chemical reduction (e.g., with H_2_, NaBH_4_) then produces the NPs settled in the body of the MOF. This method was successfully used to prepare mono- and bimetallic NPs inside MOFs (e.g., Pt NPs [161,162,163,164] and Pt/Ni NPs [165]). In addition, the chemical vapor deposition (CVD) technique was found to be advantageous in the deposition of metal nanoparticles in MOFs because, during the CVD vapor phase, the precursor is loaded in MOFs, and the precursor is then decomposed or reduced to obtain nanoparticles inside the MOF pores. Using the CVD method, MOF-5 was first infiltrated with Cu, Pd, and Au NPs in 2005 [166]. Ten years later, a new method of NP incorporation inside MOFs was developed based on encapsulation (that is, not infiltration) of the pre-synthesized NPs by growing MOF around them (Figure 10c) [167,168]. It is very important that the MOF cavities remain unharmed, and this kind of material can provide a synergistic function that is derived from both NPs and MOFs [169]. In this case, the metal NPs are completely enclosed in the so-called “metal@MOF” [170]. The interfacial region between MOFs and metal NPs plays an important catalytic role in the improvement of gas adsorption. In addition, because of a special core/shell design, MOFs can act as a partition between the NPs to prevent their sintering during the reaction, resulting in high durability.

Palladium was among the first examples of metal NPs inside MOFs. For example, Pd NPs (with an average size of 2.5 nm) were successfully infiltrated in MIL-100(Al) with a high metal content (10 wt%) without degradation of MOF [171]. As a result of Pd impregnation, the reduction in the *n_σ_* value at 77 K and 40 bar of H_2_ was directly related to the decrease in the *A_s_* and *V_a_* values (from 380 to 1200 m^2^·g^–1^, and from 0.33 to 0.65 cm^3^·g^–1^, respectively), whereas at 298 K, the *n_σ_* value increased, most probably because of the Pd hydride formation. Formation of PdH_0_._6_ was experimentally confirmed for the Pd NPs incorporated in MIL-100(Al), although the spill-over effect was also considered. As a result of the spill-over effect, faster kinetics was experimentally observed for Pd NPs encapsulated in HKUST-1 (Cu(II) 1,3,5-benzenetricarboxylate) [169]. A lower field shift in solid state ^2^H NMR suggested that relatively large amounts of hydrogen were located in the Pd lattice of Pd@HKUST-1 than those of the pure Pd NPs. Additionally, XPS measurements showed that Pd 3d binding energies of Pd@HKUST-1 were shifted to higher energy, suggesting that Pd was in a partial oxidization state. In contrast, the Cu 2p binding energies of Pd@HKUST-1 were shifted to lower energy, suggesting a partial reduction in Cu^2+^. Based on XPS data, it was concluded that the electrons in the Pd NPs were slightly transferred to HKUST-1, and this electron transfer may be accountable for the increased number of holes in the 4d band of Pd NPs encapsulated in HKUST-1, resulting in a higher amount of H_2_ adsorption. Pd NPs with a size of 1 nm were successfully infiltrated inside MIL-101 up to 20 wt% [172]. Experimentally, it was confirmed that during H_2_ absorption Pd NPs with a size of 2–3 nm formed a hydride phase (as in the case of bulk Pd), whereas Pd NPs with a size of 1 nm created solid solutions under ambient conditions. Additionally, it was concluded that because of the size effect, a decrease in the critical temperature of the two-phase region below room temperature occurred, and the *E_a_* value of H_2_ desorption for Pd NPs was lower than that of bulk Pd. The authors of [173] attempted to introduce Pd NPs inside the [Al(OH)BPDC] (BPDC = 4, 4′-biphenyldicarboxylic acid) framework, which is well known as DUT-5, using a solution impregnation procedure, as in [171]. The structure of DUT-5 was partially destroyed because of acid treatment during Pd NP infiltration, and this was the main reason for lower H_2_ adsorption compared with pristine DUT-5. Nevertheless, the higher content of Pd NPs (from 1% to 5%) promoted enhanced H_2_ adsorption at 298 K and 50 bar of H_2_, which was explained by the spill-over effect. Using theoretical predictions, the authors of [174] infiltrated Pd NPs in the pores of Br-UiO-66 and confirmed the strong host–guest interactions that keep Pd NP inside MOF. It was concluded that only monofunctionalized linkers (e.g., 2-bromoterephthalate or 2-aminoterephthalate) allowed intercalation of Pd NPs in the MOF pores, which was in contrast to the unfunctionalized (terephthalate) or biofunctionalized (2,5-dichloroterephthalate and 2,5-dihydroxylterephthalate) linkers. Additionally, it was summarized that the Pd NPs were maintained in the pores of MOFs with different functionalities, and accordingly had different degrees of interaction with the frameworks. This approach was proposed as a means of altering the catalytic activity of the metal NPs.

Platinum is another attractive example of the incorporation of metal NPs in MOFs. In 2010, two independent research groups studied MOF-5 doped with Pt NPs [175,176]. They prepared composites as physical mixtures and connected MOF-5 with Pt catalysts (5 and 10 wt%) via carbon bridges. The authors experimentally found a correlation between hydrogen adsorption and BET surface area, suggesting a typical physisorption process [175] and that the structure of the composites was similar to that of pristine MOF-5 [176]. The measured H_2_ adsorption of the carbon-bridged MOF-5 with Pt NPs was even less than that of Pt NPs on activated carbon [175]. A decrease in H_2_ uptake for the composites compared to pure MOF-5 was found in [176] and attributed to degradation of the textural properties. Moreover, no hydrogen spillover effects were found in [175,176]. On the contrary, due to the incorporation of Pt NPs in IRMOF-8 [177] and MOF-177 [178], an increase in H_2_ adsorption was experimentally observed. For Pt-modified IRMOF-8 prepared by CVD, the dependence of hydrogen adsorption capacity on the size of Pt NPs was indicated as Pt/IRMOF-8-1 > Pt/IRMOF-8-2 > Pt/IRMOF-8-3, where the incorporated Pt NPs had sizes of 2.2, 3.9, and 9.1 nm, respectively. This indicates that, depending on the pore’s size in MOFs, surface decoration or bulk infiltration with NPs may be possible; therefore, the geometry of both NPs and MOFs should be taken into account. Additionally, it was concluded that the Pt NPs embedded in MOFs may adsorb and absorb H atoms to hydrogenate Pt to a stable metal hydride. This happened, for example, for Pt@MOF-177 [178]. The encapsulated Pt NPs in MOF-177 showed H_2_ adsorption of 2.5 wt% at 298 K and 144 bar of H_2_ in the first adsorption cycle, but only 0.5 wt% in the second cycle, which was similar to the value of pure MOF-177. Therefore, to avoid direct hydrogenation of the metal NPs incorporated in MOFs, the platinum group metals should be changed to transition metals to ensure they do not react readily with atomic hydrogen.

**Figure 11:**
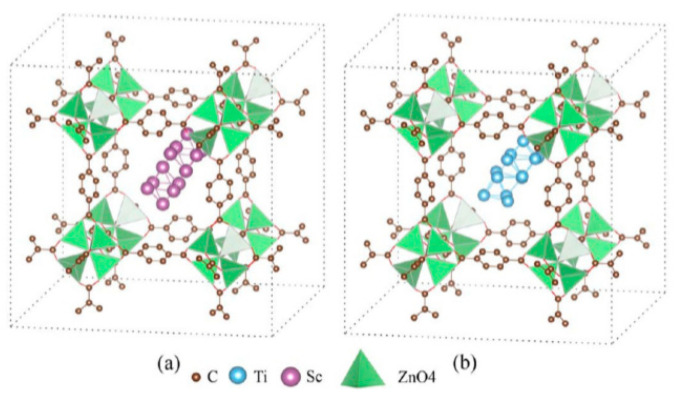
Optimized geometry of MOF-5 in which a cluster of 12 metal atoms of Sc (**a**) or Ti (**b**) is relaxed inside the pore. Reprinted with permission from [179]. Copyright © 2012, American Chemical Society. All rights reserved.

Scandium and titanium may be considered as promising candidates for their incorporation as NPs in MOFs. In the theoretical work [179], the effect of these light transition metals on the hydrogen adsorption in MOF-5 was calculated using the ab initio density functional theory (DFT). The calculations predicted that Ti atoms may undergo clustering (Figure 11), whereas Sc atoms preferred the full metal decoration of MOF-5. Both Sc- and Ti-decorated MOF-5 may adsorb the 5H_2_ molecules per metal atom, resulting in a theoretical hydrogen adsorption capacity of 5.81 and 5.72 wt%, respectively. The calculated *Q_st_* value was predicted within the range of 20–40 kJ·mol^–1^ and additional improvement in the interaction energy between the metals and MOF-5 may be achieved with boron substitution.

Successful preparation of Ni NPs in Ni-MOF-74 was carried out with a partial thermal decomposition of the same MOF [180]. The Ni_2_(dhtp) (H_4_dhtp = 2,5-dihydroxyterephthalic acid) framework is characterized by pores with a size of ~11 Å and integrated from the Ni^2+^ nodes, which can be chemically reduced by organic linkers (hydroqinone). In this thermal method, the size of the intercalated Ni NPs can be altered with temperature values. Surface decoration with the NiNPs was made for MIL-100, and the resulting material showed a high catalytic activity for hydrogen evolution reaction (HER) [181]. Then, the NiMo alloy was introduced into MIL-101 and the prepared NiMo@MIL-101 demonstrated greater HER catalytic activity than that of Ni@MIL-101 [182]. Ni NP intercalation in MOF-5 was carried out in [183] and the synthesized Ni@MOF-5 was tested as a catalyst in the production of H_2_ from water splitting under visible light irradiation. The Ni-modified MOFs were widely investigated in terms of their catalytic and optical properties [184] but were not considered to be an effective porous material for H_2_ adsorption. Development of the preparation methods to incorporate metal NPs in MOFs began several years ago, but the isosteric enthalpy of their hydrogen adsorption and hydrogen adsorption capacity has not been fully examined. In addition, the origin of the enhanced H_2_ adsorption, and the mechanism of interactions between metal NPs and MOFs, remain unclear.

#### 4.3.2. Morphology of Pores and Their Functionalization

The effect of the pore size may be explained by the fact that the small diameter of pores enables energy potentials to overlap between the opposing walls, resulting in higher interaction energy with H_2_ molecules [185]. For example, MOFs of M(HBTC) (4,4′-bpy)·3DMF (M = Ni, Co; HBTC = 1,3,5-benzenetricarboxylic acid; 4,4′-bipy = 4,4′-bipyridine; DMF = N,N′-dimethylformamide) showed H_2_ adsorption of 3.42 and 2.05 wt% at 77 K and 1 bar of H_2_, and 1.20 and 0.96 wt% at 298 K and 70 bar of H_2_, respectively [186]. The *A_s_* values measured by the BET method were 1590 and 887 m^2^·g^–1^, respectively, but it was noted that these two frameworks had nonlinear rectangular channels with an average size of 7 × 6 Å. Another [Co_3_(NDC)_3_(dabco)] (dabco = 1,4-diazabicyclo[2.2.2]octane) framework with a primitive cubic structure demonstrated 0.89 wt% of H_2_ at 298 K and *P*(H_2_) = 17.2 bar, which was explained by both the *A_s_* value (1502 m^2^·g^−1^) and the average pore diameter (4.5 Å) [187]. The four-fold interpenetrating networks based on the [OZn_4_]^6+^ nodes with 3D channels (<5 Å) might also be considered. These examples indicate that, even with a small *A_s_* value, hydrogen adsorption can be significant and, most importantly, controlled by pore size (5–7 Å).

**Figure 12:**
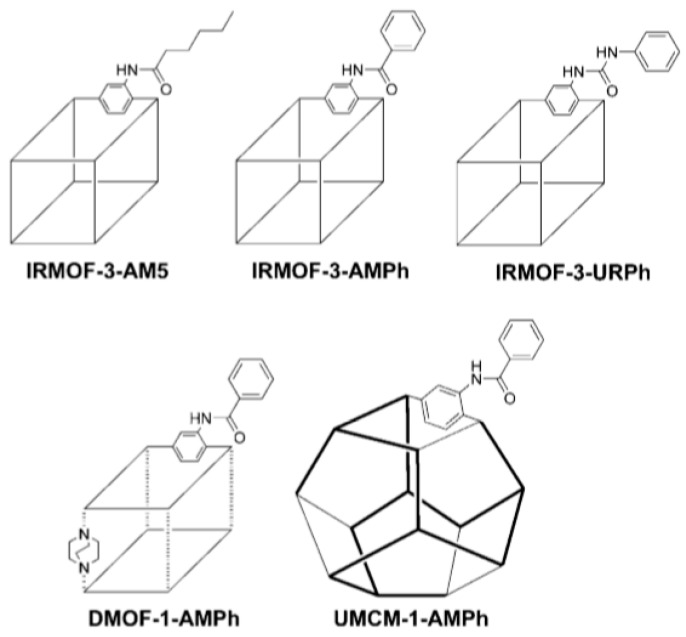
Schematic representation of pore functionalization by amide groups in IRMOF-3-AM5, IRMOF-3-AMPh, IRMOF-3-URPh, DMOF-1-AMPh, and UMCM-1-AMPh. Reprinted with permission from [188]. Copyright © 2010, Wiley-VCH Verlag GmbH&Co. KGaA, Weinheim. All rights reserved.

It is clear that the morphology of pores, in addition to the chemical environment of all of the pore walls, can be adjusted with functionalization of the internal surface of MOFs. The value of this functionalization has also been considered to be a possible impact on H_2_ adsorption. The authors of [188] attempted to change the pore morphology of MOFs to improve the *Q_st_* value and hydrogen adsorption capacity. They reacted IRMOF-3, DMOF-1-NH_2_, and UMCM-1-NH_2_ with anhydrides or isocyanates to obtain IRMOF-3-AM5, IRMOF-3-AMPh, IRMOF-3-URPh, DMOF-1-AMPh, and UMCM-1-AMPh with pore functionalization by amide groups (Figure 12). It was experimentally confirmed that the post-synthetic modification resulted in higher H_2_ adsorption, especially when pore functionalization was undertaken with phenyl ring substituents. This supports the suggested role of aromatic rings on hydrogen adsorption presented in Section 4.1.1.

The authors of [189] constructed functional porous MOFs via the reaction of Ni^2+^ ions with 2,4,6-tri(4-pyridinyl)-1,3,5-triazine (tpt) and *o*-phthalic acid as a co-ligand. It was concluded that the decoration of the pores’ surface and tuning of adsorption properties could be achieved by introducing different functional groups on the *o*-phthalic acid ligand; therefore, a new class of TKL frameworks (TKL = Tianjin Key Lab of Metal and Molecule Based Materials) with different functionalized (–NH_2_, –NO_2_, and –F) *o*-phthalic acid was studied. It was determined experimentally that all of the fluorinated MOFs with different numbers and positions of substituted F-atoms demonstrated high values of *A_s_* and H_2_ adsorption. At 65 bar of H_2_ and 77 K, hydrogen adsorption for TKL-106 and TKL-107 was estimated to be 6.24 and 4.00 wt%, respectively. Due to pore functionalization, greater interaction between H_2_ molecules and the fluorinated MOFs was explained by the micropore diameter (approximately 6.0 Å), which is very close to the approximate size of two H_2_ molecules (2 × 2.8 Å).

In the experimental work [190], the effect of solvent used in MOF-5 preparation on pore morphology was studied. Narrow slit-like pores with internal irregular voids were observed in the synthesized MOF-5 with coexisting micro-, meso-, and macropores. For meso- and macroporous regions, the values of hydrogen *Q_st_* were calculated as 3.68 and 12.45 kJ·mol^–1^, respectively. The higher value was explained by the presence of a residual solvent in macropores, which increased interactions between H_2_ molecules and pore walls.

## 5. Prospective Views and Outlooks

MOFs are unique chemical compounds that can be used in numerous scientific applications, including hydrogen adsorption; however, adapting a pure MOF to the DoE target to be used as an effective hydrogen storage material remains a significant challenge. The factors of surface area and pore volume remain the most critical issue, because at 298 K, the *n_a_* value correlates mainly with the *V_a_* value at low *P*(H_2_) and the *A_s_* value at high *P*(H_2_). Organic linkers have a direct influence on the pore morphology, and therefore, on the surface area of MOFs, their length, chemical composition, and flexibility may explain the catenation and sponge-like dynamic mechanism in MOFs. These effects may change the hydrogen adsorption in pure MOFs, but their absolute quantity of adsorbed hydrogen does not meet the DoE requirements at 298 K, or at 100 bar of H_2_. The value of *Q_st_* may be considered to be a reasonable indicator of the applicability of pure MOFs as a hydrogen storage material. At 298 K, a tendency exists to form a proportional relationship between the *Q_st_* and the *n_σ_* values, and using the approach of open metal sites or an electrostatic field, isosteric enthalpy of hydrogen adsorption increases slightly in pure MOFs. Numerous calculations suggest with values of *Q_st_* within the range of 15–25 kJ·mol^–1^, with a *V_a_* value of ~2.5 cm^3^·g^–1^ and porosity of ~85%, would be desirable for an MOF as a successful candidate for hydrogen storage with 9 wt% of H_2_.

Nanoscaffolding materials based on MOFs, sometimes generalized to “hybrid” or “functional” composites, may combine properties of their components. In practice, isosteric enthalpy of hydrogen adsorption and hydrogen adsorption capacity has not been fully examined for this kind of material, and is not reproducible for the same examples. The main reasons for these discrepancies may lie in the morphology of pores and their functionalization, which is highly sensitive to the synthesis procedure and post-synthetic modification of MOFs. Another approach to nanoscaffolding materials based on MOFs may be nanoconfinement of metal hydrides inside the frameworks, in which MOFs are considered to be nanoscaffolds rather than H_2_ sorbents. The main advantages of nanoconfined hydrides relate to their faster kinetics, higher reversibility, and the change in the mechanism of hydrogen interaction. The additional weight of nanoscaffolds leads to lower gravimetric hydrogen sorption capacity in the composite material. Recently, MOFs characterized by the morphology of their pores and functionalization were considered to be suitable microporous scaffolds. For example, the Ti-modified NaAlH_4_ infiltrated in MOF-74(Mg) was successfully synthesized by melt infiltration, achieving loadings up to 21 wt% [191]. MOF-74(Mg) characterized by pores with a diameter of 12 Å and lined with Mg atoms have open coordination sites, which were considered to be sites for TiCl_4_ stabilization. In practice, nanoconfinement of NaAlH_4_ has indicated advantages with respect to the bulk analogue (Table 4) [191]. The first, typical decomposition pathway for bulk NaAlH_4_ was simplified by avoiding the stable Na_3_AlH_6_ intermediate production. Secondly, kinetics of decomposition for nanoconfined NaAlH_4_ was higher, as confirmed by lowered values of the T_dec_(NaAlH_4_) and decreased values of activation energy (*E_a_*). Titanium modification considerably increased the reversibility of the NaAlH_4_ decomposition-formation. These obtained results were explained by chemical and structural features of MOF-74(Mg), which was used as an effective nanoscaffold for NaAlH_4_. Micropores of MOF-74(Mg) allow NaAlH_4_ to be in close contact with the pore walls; hence, the chemical environment of all of the pore walls can stabilize the final decomposition products (including H_2_ molecules), avoiding their diffusion and agglomeration.

In another example, highly reactive gaseous Ti(BH_4_)_3_ molecules at 298 K were infiltrated into UiO-66 [192]. The nanoconfined hydride was stabilized in the porous MOF up to 350 K under vacuum and reached approximately double gas density. After Ti(BH_4_)_3_ infiltration, the value of UiO-66 obtained by the BET method decreased from 1200 to 770 m^2^·g^−1^. The nanoconfined Ti(BH_4_)_3_ demonstrated another mechanism of decomposition, including the production of pentaborane rather than diborane in the case of pure Ti(BH_4_)_3_. These experimental results encourage the development of other examples of nanoconfinement, including solid hydrides with a high hydrogen content and stable MOFs with pore characteristics suitable for effective nanoscaffolding.

To optimize the hydrogen adsorption process, data are required regarding the physicochemical characteristics of MOFs to better understand their interaction with H_2_ molecules. To obtain such important information, vibrational spectroscopy plays a crucial role and is often notable in many cases. At present, IR and Raman spectroscopies are considered to be powerful tools for initial characterization of MOFs and their composites, including in situ measurements under hydrogen adsorption, in addition to their combinations with other techniques to study crystal structure and morphology, and physical and chemical properties of MOFs [193].

## 6. Conclusions

Hydrogen sorption is a complex physical and chemical process, which includes both adsorption at the surface and absorption into the bulk. Different hydrogen storage material MOFs may represent an opportunity to study the molecular hydrogen interaction with microporous crystalline materials made of metal nodes and organic linkers. The main benefit of MOFs relates to their reversible and high-rate hydrogen adsorption process, whereas their biggest disadvantage is their operation at very low temperatures. Using the factors of surface area, pore volume, and isosteric enthalpy of hydrogen adsorption, storage of hydrogen gas in MOFs may be increased. The catalytic effect and pore design are anticipated to be additional influences on hydrogen adsorption because of the incorporated catalyst inside the MOFs and their pore functionalization. Nanoscaffolding materials, in which MOFs are considered to be nanoscaffolds, rather than H_2_ adsorbents, may be considered to be a suitable nanotechnology to achieve DoE conditions when this approach can be used to reduce the gravimetric hydrogen storage capacity.

## Figures and Tables

**Table 1:** Specific surface area, pore volume, and H_2_ adsorption for structurally modified MOF-5. Adapted with permission from [84]. Copyright © 2012, Elsevier. B.V. All rights reserved.

MOF-5 Sample	*A_s_*, m^2^·g^–1^	*V_a_,* cm^3^·g^–1^	Pore Size, Å	wt% of H_2_	*T*_dec_, K
Low-crystalline	2050	0.88	~6 and ~13	1.2	711
High-crystalline	3400	1.42	~7 and ~14	1.3	755
Interwoven	1010	0.51	~6	1.7	773
Interwoven with incorporated MWCNTs	1170	0.57	~6	2.0	783

**Table 2:** Surface area (BET), pore volume, and H_2_ adsorption capacity at 77 K and 1 bar of H_2_ for IRMOFs.

MOF Sample	Organic Linker	Pore Characteristics		H_2_ Adsorption, wt%
		*A_s_*, m^2^·g^–1^	*V_a_,* cm^3^·g^–1^	
IRMOF-1	1,4-benzenedicarboxylate	3362	----	1.32
IRMOF-2	2-bromobenzene-1,4-dicarboxylate	1722	0.88	1.21
IRMOF-3	2-aminobenzene-1,4-dicarboxylate	2446	1.07	1.42
IRMOF-6	1,2-dihydrocyclobutabenzene-3,6-dicarboxylate	2476	1.14	1.48
IRMOF-8	Naphthalenedicarboxylate	890	0.45	1.45
IRMOF-9	4,4′-biphenyldicarboxylate	1904	0.9	1.17
IRMOF-11	4,5,9,10-tetrahydropyrene-2,7-dicarboxylate	----	----	1.62
IRMOF-13	pyrene-2,7-dicarboxylate	1551	0.73	1.73
IRMOF-16	p-terphenyl-4,4′-dicarboxylate	----	----	----
IRMOF-18	2,3,5,6-tetramethylbenzene-1,4-dicarboxylate	1501	----	0.89
IRMOF-20	thieno[3,2-b]thiophen-2,5-dicarboxylate	3409	1.53	1.35

**Table 3:** Values of surface excess and the total adsorbed amount of hydrogen (experimentally obtained at 77 or 298 K and 50 bar of H_2_) for catenated PCN-6 and non-catenated PCN-60.

Temperature, K	Catenated PCN-6		Non-Catenated PCN-60	
	*n_a_,* wt% of H_2_	*n_σ_*, wt% of H_2_	*n_a_*, wt% of H_2_	*n_σ_*, wt% of H_2_
77	8.7	6.7	5.5	4.0
298	1.5	0.9	0.8	0.4

**Table 4:** Hydrogen adsorption capacity and activation energy of decomposition for nanoconfined and bulk NaAlH_4_. Hydrogen adsorption capacity was measured at 433 K and 105 bar of H_2_, and the mass of MOF-74(Mg) was excluded from the determination of the hydrogen amount. Reprinted with permission from [191]. Copyright © 2012, American Chemical Society. All rights reserved.

NaAlH_4_	Amount of Ti, mol%	*E_a_*, kJ·mol^–1^	H_2_ Adsorption, wt%
Bulk	0	118.1	5.12
Bulk	2	79.5	4.25
Infiltrated in MOF-74(Mg)	3	57.4	4.20
